# Protein-protein interactions in plant antioxidant defense

**DOI:** 10.3389/fpls.2022.1035573

**Published:** 2022-12-14

**Authors:** Pavol Melicher, Petr Dvořák, Jozef Šamaj, Tomáš Takáč

**Affiliations:** Department of Biotechnology, Faculty of Science, Palacký University, Olomouc, Olomouc, Czechia

**Keywords:** plants, antioxidant enzymes, protein-protein interactions, stress response, reactive oxygen species, receptor for activated C kinase 1

## Abstract

The regulation of reactive oxygen species (ROS) levels in plants is ensured by mechanisms preventing their over accumulation, and by diverse antioxidants, including enzymes and nonenzymatic compounds. These are affected by redox conditions, posttranslational modifications, transcriptional and posttranscriptional modifications, Ca^2+^, nitric oxide (NO) and mitogen-activated protein kinase signaling pathways. Recent knowledge about protein-protein interactions (PPIs) of antioxidant enzymes advanced during last decade. The best-known examples are interactions mediated by redox buffering proteins such as thioredoxins and glutaredoxins. This review summarizes interactions of major antioxidant enzymes with regulatory and signaling proteins and their diverse functions. Such interactions are important for stability, degradation and activation of interacting partners. Moreover, PPIs of antioxidant enzymes may connect diverse metabolic processes with ROS scavenging. Proteins like receptor for activated C kinase 1 may ensure coordination of antioxidant enzymes to ensure efficient ROS regulation. Nevertheless, PPIs in antioxidant defense are understudied, and intensive research is required to define their role in complex regulation of ROS scavenging.

## Introduction

Reactive oxygen species (ROS) are integral regulatory components of multiple metabolic and developmental pathways under normal and stress conditions in plants. Due to the ability to cause harmful irreversible oxidative modifications of biomolecules, ROS cellular levels must be controlled by precisely orchestrated antioxidative systems. These include effective nonenzymatic antioxidants and array of antioxidant enzymes compartmentalized into the main subcellular sites of ROS production (Mittler et al., 2022). Mechanisms regulating antioxidant enzymes are multifaceted and involve retrograde signaling, redox modifications, transcriptional, posttranscriptional and posttranslational regulations (Dvořák et al., 2021b). Another level of regulation is represented by the interaction of antioxidant enzymes with diverse proteins. Among the most important interactions in redox biology are those occurring between redox buffering proteins such as thioredoxins and glutaredoxins and their targets to reverse ROS- and NO-mediated redox modifications of target proteins (Mittler et al., 2022). Nevertheless, compelling evidence exists about protein-protein interactions (PPIs) of antioxidant enzymes with regulatory proteins such as activators, chaperones, stabilizers, but also signaling and scaffold proteins. Employing these interactions, antioxidant enzymes take part in diverse metabolic, signaling and developmental processes and transfer signals from redox homeostasis to metabolism.

Here we summarize the current knowledge about PPIs of most important antioxidant enzymes. In addition, we provide brief overview of methods for PPI identification and describe structural details of PPIs, mainly those determined by protein redox modifications.

## Chemistry of PPIs

The ability to interact with other proteins is one of the basic attributes of proteins. Many cellular processes, including metabolism and signaling, are regulated by proteins operating in protein complexes (Nussinov et al., 2013; Sudha et al., 2014). Proteins undergo either obligatory or non-obligatory PPIs. While obligatory interactions are typical for proteins that are not functional in their protomer form, non-obligatory interactions are quite common for proteins that are active also in spatially separated form (Nooren and Thornton, 2003; Seychell and Beck, 2021). In permanent interactions, protein-protein interfaces are often hydrophobic, formed by aromatic amino acid groups and hydrophobic non-polar groups that intersperse Van der Waals bonds. In non-obligatory and transient complexes, interfaces are formed by polar and charged amino acid groups and they contain a larger number of hydrogen bonds between interaction partners (Moreira et al., 2007; Perkins et al., 2010). In addition, for transient interactions, it was found that up to 75% of amino acids in the interface participate in inter- and intra-protein interactions, which reduces the frequency of structural changes of proteins. These interactions are mediated not only by polar amino acids (Asp, Glu, His, Arg) but also by hydrophobic ones (Leu, Phe, Try, Met; Jayashree et al., 2019). Interaction interfaces in transiently interacting proteins are less conserved and exhibit higher structural plasticity as those in permanently interacting proteins (Mintseris and Weng, 2005; Levy, 2010). It is estimated that 15-40% of PPIs are mediated by short linear peptide sequences (SLiMs) with a length of 3-10 amino acids. The ability to mediate interactions can be affected by reversible post-translational modifications (PTMs) of amino acid residues, which usually spatially interfere with the protein-binding domain (Blikstad and Ivarsson, 2015; Duan and Walther, 2015). Phosphorylation, acetylation, or glycosylation are often responsible for the establishment or disruption of PPIs (Ngounou Wetie et al., 2013) by changing the electrostatic or structural properties of the interaction interface (Duan and Walther, 2015). For example, phosphorylation of Tyr32 and Tyr64 of K-Ras4B GTPase alters the binding free energy of residues in the interaction interface, thus leading to generation of fewer inter-molecular hydrogen bonds and salt bridges between Ras and its interaction partner named guanine nucleotide exchange factor son of sevenless (SOS; Wang et al., 2021). Another example shows that phosphorylation of Thr37 and Thr46 residues in intrinsically disordered protein 4E-BP2 promotes formation of four-β-stranded domains by folding in Pro18-Arg62 region, and this is weakening the interaction with eukaryotic translation initiation factor 4E (eIF4E; Bah et al., 2015). In some cases, the residue subjected to PTM may be a part of a specific motif that is recognized by conserved binding modules (Yang, 2005). One of the first explored examples of a binding module is Src homology 2 (SH2) domain, which specifically recognizes phosphorylated Tyr (pTyr) residues that also form a docking site for interaction (Hubbard and Till, 2000). In addition, there are six different classes of SH2, containing proteins that recognize pTyr but also three to five C-terminal residues neighboring pTyr, collectively contributing to SH2 module specificity (Stein et al., 2009). It has also been shown that PPIs are affected by the oxidation state of a cysteine located in the proximity of the binding site (Shi et al., 2021; van Dam et al., 2021). Cysteine oxidation may lead to disruption of interaction as shown for PPI between B-cell lymphoma (Bcl-2) and extracellular signal-regulated kinases 1/2 (ERK1/2), where oxidations of Cys158 and Cys229 on Bcl-2 by hydrogen peroxide (H_2_O_2_) cause dissociation of ERK and promotion of apoptosis in human lung epithelial cells (Luanpitpong et al., 2013). Complexes constituted by intermolecular disulfide bonds might depend on the cellular redox homeostasis. Under control conditions, salicylic acid (SA) receptor nonexpresser of PR genes 1 (NPR1) is in the form of an oligomer with individual subunits bound by intermolecular disulfide bonds through Cys82 and Cys216 (Mou et al., 2003). After pathogen attack, these bonds are broken by thioredoxin (TRX3 and TRX5)-mediated reduction, NPR1 monomer is released and triggers systemic acquired resistance (SAR) in the nucleus by interacting with transcription factor GACG sequence specific binding protein 1 (TGA1), which activates expression of defense-related genes (Mou et al., 2003; Lindermayr et al., 2010). Additionally, S-nitrosylation of Cys156 supports formation of oligomers, and thus maintains long-term NPR1 homeostasis (Tada et al., 2008). Finally, TGA1-NPR1 interaction is sensitive also to oxidative modifications of TGA1. Intramolecular disulfide bond between Cys260 and Cys266 in TGA1 prevents interaction with NPR1. SA accumulation leads to their reduction, allowing interaction with the monomeric form of NPR1 (Després et al., 2003). These findings clearly point to the role of Cys redox state and its PTM in the regulation of PPIs.

## An overview of techniques identifying PPIs

The identification of PPIs relies on multiple *in vivo* or *in vitro* techniques differing in technical complexity and throughput. The most widely used low throughput methods include yeast two-hybrid assay (Y2H), bimolecular fluorescence complementation assay (BiFC) and Förster resonance energy transfer (FRET).

Yeast two-hybrid assay (Y2H) is relatively cheap, simple and fast method suitable for studying weak and transient interactions (Vinayagam et al., 2010). It is used not only for studying the interaction of a selected pair of proteins, but also for high-throughput screening of a cDNA library to reveal new interaction partners of the protein of interest (POI; Chepelev et al., 2008). However, it suffers from a high occurrence of false positive and false negative interactions (Sprinzak et al., 2003) and the inability to study membrane proteins. In addition, the heterologous system of yeast limits the studies of the PTM-dependent interactions in plants (Ngounou Wetie et al., 2019) and cannot reveal the indirect interaction of proteins in a multiprotein complex. It has to be noted that the drawbacks of Y2H have been improved by various modifications (Xing et al., 2016).

BiFC detects the interaction of two proteins, each of them being fused to one of the complementary terminal fragments of fluorescent protein (FP). If the proteins interact, complementary reconstitution of the reporter FP occurs and may be detected by fluorescence microscopy (Hu et al., 2002; Walter et al., 2004). This method enables the study of transient PPIs and provides information on the subcellular localization of the interaction. Nevertheless, the high affinity of complementary FP fragments may result in FP reconstitution without interaction of analyzed proteins, especially in cases of their overabundance. The high stability of reconstituted FP prevents studying dynamic interactions. On the other hand, the irreversibility of this complex allows identification of weak and transient interactions (Kerppola, 2006; Cui et al., 2019). BiFC has also been applied for high-throughput analyses (Miller et al., 2015).

Another widely applied *in vivo* method of studying PPIs is based on FRET (Förster, 1948). The principle of this low-throughput method is based on the fusion of two POIs to suitable donor or acceptor fluorophores or FPs, also called FRET pairs, which can undergo a nonradiative (dipole-dipole) transfer of energy if they are in distance less than 10 nm from each other, and detection of emission shift from donor to acceptor by confocal microscopy. The main advantage of FRET is that it enables spatio-temporal interaction studies in living cells with high precision and resolution (Cui et al., 2019; Struk et al., 2019). However, classical FRET analysis suffers from the presence of high background caused by spectral bleed-through and autofluorescence (Xing et al., 2016). Therefore, several FRET modifications suitable for *in vivo* studies of PPIs have been developed, such as FRET-FLIM (fluorescence lifetime imaging microscopy), FRET-APB (acceptor photobleaching), Triple-FRET, Homo-FRET and others (Xing et al., 2016; Cui et al., 2019; Strotmann and Stahl, 2022).

Proximity labelling (PL) is an *in vivo* high throughput approach that allows to study weak, transient or hydrophobic PPIs in living cells using enzyme-dependent labelling of proteins that are in the close proximity to POI and their subsequent MS analysis (Qin et al., 2021; Yang et al., 2021). A catalytic enzyme is fused to the POI and converts a suitable substrate into a reactive product that binds to proteins in the POI proximity. The substrate often contains biotin moieties which allows fast isolation and enrichment of labelled proteins from the mixture. Recently, a new method for PPI in cell lines based on PL was proposed (McCutcheon et al., 2020). POI is first modified by a photoproximity label in living cells. After light-triggered photocleavage, covalent labelling of proximal proteins occurs by reactive carbene of the released label. The advantage of this method is that it allows studying PPIs of redox-sensitive proteins, which are highly dependent on the redox state of the cell. This method was designed and used in the study of kelch-like ECH-associated protein 1 (KEAP1) in the HEK293T cell line so far and confirmed the presence of known but also new KEAP1 interactors (McCutcheon et al., 2020).

Co-fractionation coupled with mass spectrometry (CF-MS) is a high-throughput method allowing the detection of interacting proteins in native non-denaturing conditions, without the need for antibodies or epitope tagging of studied POI. Native protein extract is separated chromatographically and proteins in each biochemical fraction are identified by MS. PPI is confirmed when co-elution of interacting proteins occurs over multiple distinct separations (Wan et al., 2015; McWhite et al., 2020).

Affinity purification (AP) of protein complexes is suitable for PPI detection *in vitro*, both on low and high throughput levels. It reduces the complexity of the sample and allows the purification of protein complexes prior to MS analysis. It is based on co-purification of native or epitope-tagged POI and its interaction partners and subsequent MS/MS analysis of the eluted complex (Gingras et al., 2007). Tandem AP-MS (TAP-MS) is an approach which combines two different tags fused to POI in tandem divided by cleavage site, allowing two-step purification and reducing false positive interactions (Rigaut et al., 1999; Leene et al., 2008). The most high-throughput methods are prone to false-positive results, and it is assumed that more than half of all high-throughput data are not correct (von Mering et al., 2002; Sprinzak et al., 2003). AP-MS often leads to a detection of larger amounts of proteins that form the negative background and thus impair the reliability of the analysis. This problem is partially solved by TAP-MS, the disadvantage of which, however, is the loss of weak and transient interactions (Gavin et al., 2011). Noteworthy, even a well performed AP/TAP-MS-based PPI analysis needs to be verified. Co-immunoprecipitation (Co-IP) together with Western blotting (WB) are most frequently used methods to validate AP/TAP-MS data (Ngounou Wetie et al., 2013). Another way to confirm interaction is by reciprocal isolation, in which the role of bait and target proteins are switched and reciprocally analyzed (Miteva et al., 2013). The interaction probability might be increased by additional bioinformatic analyses, such as prediction of localization, PPI probability and co-expression analysis.

Currently, a reliable identification of PPI requires the application of at least two (but preferably three) independent techniques (Xing et al., 2016; Vadovič et al., 2019; Samakovli et al., 2020). Each method suffers from shortcomings that lead to false-positive and false-negative results, and it is therefore necessary to use proper positive and negative controls.

It is rather challenging task to isolate and identify interactors of redox-sensitive proteins or interactions conditioned by their oxidative state. Such studies are mostly based on above-mentioned methods with various modifications. An effective approach is the substitution of the putative regulatory/binding redox modified residues followed by correlation of the interaction between native and mutated form of the protein under Cys-modifying conditions. Using this principle, the interactions of *Arabidopsis* SKP1-like1 (ASK1) with cullin 1 (CUL1), transport inhibitor resistant 1 (TIR1) coronatine insensitive 1 (COI1) and auxin signaling F-Box (AFB) of the E3 ubiquitin ligase complex SKP1–cullin–F-box^TIR1/AFBs^ (SCF^TIR1/AFBs^; Iglesias et al., 2018) have been identified and characterized. It was found that ASK1 Cys118 and Cys37 can undergo S-nitrosylation or S-glutathionylation. Employing Y2H (using sodium nitroprusside as the NO donor) and pull-down analyses (using NO-Cys as the nitrosylating agent) with mutated versions of ASK1 (C118A and C37A) it was revealed that the interaction with the SCF^TIR1/AFB^ or the SCF^COI1^ complex proteins depends on S-nitrosylation of these Cys residues and leads to disruption of auxin and jasmonic acid (JA) signaling in *Arabidopsis* (Iglesias et al., 2018; Terrile et al., 2022).

An efficient approach was used during the study of the redox-dependent interactome of 2-cysteine ​​peroxiredoxins (2-CysPRXs) in *Arabidopsis*. The redox conformation-specific 2-CysPRX interactome was investigated using affinity pull-down (Liebthal et al., 2020). Authors have used site-targeted mutated pseudoreduced C54S and pseudohyperoxidized C54D variants to reveal interactomes in oxidized and reduced leaf cells lysates (König et al., 2013; Liebthal et al., 2020).

Redox sensitive interactome may be also studied using H_2_O_2_-oxidized and DTT-reduced forms of tagged POIs by pull-down assay. Interactome of different redox forms of phosphatase and tensin homolog (PTEN) acting as a tumor suppressor protein during cancer development was studied by this approach (Verrastro et al., 2016; Chen et al., 2018). Thus, oxidized or reduced forms of GST-tagged PTEN were used as a bait and the HCT116 cell lysate was pulled-down to bind PTEN redox-sensitive interactome. Label-free LC-MS analysis was performed to identify interactors and results were validated by WB (Verrastro et al., 2016).

## PPIs of plant superoxide dismutases

SODs catalyze the dismutation of superoxide anion to less toxic H_2_O_2_, an essential component of plant abiotic and biotic stress signaling (Castro et al., 2021; Mittler et al., 2022). Plant SOD isoforms are subcellularly compartmentalized into chloroplasts, mitochondria, cytosol, peroxisomes and apoplast, the main sites of ${\text{O}}_{2^{-}}$ production. In the genome of *Arabidopsis*, three genes encoding *FeSODs* (*FSD1*, *FSD2*, and *FSD3*), two genes coding for *MnSODs* (*MSD1*; *MSD2*), and three genes encoding *Cu/ZnSODs* (*CSD1*, *CSD2*, and *CSD3*) have been described (Kliebenstein et al., 1998; Pilon et al., 2011; Dvořák et al., 2021a; Chen et al., 2022).

SODs respond to changes in external conditions *via* transcriptional, posttranscriptional and posttranslational regulation (Dvořák et al., 2021b). One of the major factors affecting expression of *FSD1* and *CSD* genes is the availability of Cu^2+^ (Yamasaki et al., 2009; Yan et al., 2017; Melicher et al., 2022). Furthermore, expression of *SOD* genes also depends on diverse transcription factors under changing external conditions (Dvořák et al., 2021b). SODs are also modulated by various PTMs such as phosphorylation, nitration, glutathionylation, and glycation (reviewed in Yamakura and Kawasaki, 2010; Banks and Andersen, 2019; Dvořák et al., 2021b). Moreover, this complex regulatory network is complemented by vital PPIs.

As experimentally confirmed by in-gel SOD activity assay, yeast Lys-independent aerobic growth assay and Y2H, *Arabidopsis* CSD isoforms are activated by interaction with copper chaperone for superoxide dismutase (CCS; **Table 1**), which delivers Cu^2+^ to their active site (Abdel-Ghany et al., 2005; Chu et al., 2005; Cohu et al., 2009; Huang et al., 2012). The activation of Cu/ZnSOD by CCS is an evolutionarily conserved process and it was confirmed for SOD1 in *S. cerevisiae* (Rae et al., 1999; Schmidt et al., 2000), mice and humans (Wong et al., 2000; Carroll et al., 2004). All three protein domains of CCS (N-terminal antioxidant protein 1 (ATX1)-like domain containing the copper-binding site, central domain responsible for physical interaction with Cu/ZnSOD, and C-terminal domain responsible for Cu^2+^ transfer to Cu/ZnSOD) show a high degree of conservation (Dreyer and Schippers, 2019), contribute to the binding activity and are essential for Cu/ZnSOD activation (Schmidt et al., 1999; Lamb et al., 2001; Chu et al., 2005). *Arabidopsis* CCS occurs in three splicing variants showing different subcellular localization. While the N-terminal plastid transit peptide-containing CCS320 provides specific activation of CSD2, cytosolic CSD1 and peroxisomal CSD3 are possibly activated by CCS184 or CCS229, which both contain C-terminal peroxisomal targeting peptide (Chu et al., 2005; Dreyer and Schippers, 2019). However, an existence of CCS-independent activation pathway was suggested for CSD1 and CSD3 (Huang et al., 2012). This is also supported by the absence of *CCS* gene in some organisms containing Cu/ZnSOD, such as *Caenorhabditis elegans* or *Drosophila melanogaster* (Jensen and Culotta, 2005; Dreyer and Schippers, 2019). The activation of CSD1 might be mediated in *Arabidopsis* by an interaction with DJ-1 homolog A (AtDJ-1A; **Table 1**) protein, as examined by isothermal titration calorimetry and BiFC (Xu et al., 2010). Genetic studies indicated the involvement of AtDJ-1A in protection against oxidative stress and high light by CSD1 activation, however AtDJ-1A may exhibit intrinsic antioxidant activity as well (Xu et al., 2010). Its human orthologue was identified as a SOD1 activating Cu^2+^ chaperone, potentially delivering Cu^2+^ to its catalytic center (Girotto et al., 2014). *At*DJ-1 protein shows several conserved residues across plants, humans and bacteria. Unlike other organisms, *Arabidopsis* exhibit duplication of *AtDJ-1*, as AtDJ-1A, B and C isoforms contain two full-length DJ-1/PARK7 polypeptides (Wei et al., 2007; Xu et al., 2010). It remains to be elucidated, whether an analogous activation mechanism also occurs for CSD2 in chloroplasts by AtDJ-1B and AtDJ-1C isoforms, which are essential for plant viability and chloroplast development (Lin et al., 2011) and have chaperone activity (Lewandowska et al., 2019). It has to be noted, that *Arabidopsis ccs* mutant entirely lacks CSD2 activity (Cohu et al., 2009; Huang et al., 2012).

**Table 1:** List of *Arabidopsis* superoxide dismutases interaction partners found by low-throughput methods with their respective function and localization.

Protein of interest	Accession	Interactor name	Function	Localization	Method of detection	Reference
FSD1/2/3	AT5G20720	CPN20, 20 kDa chaperonin	co-chaperone, FSDs activation, ABA signaling	plastid, chloroplast	in-gel activity assay (L)	Kuo et al., 2013b
					FRET^1^ (L)	
					Y2H (L)	
FSD2	AT3G49580	LSU1, protein response to low sulfur 1	response to S deficiency, plant immune responses	Unknown	Y2H (H)	*Arabidopsis* Interactome Mapping Consortium, 2011
					*in vitro* pull-down/WB (L)	Garcia-Molina et al., 2017
					BiFC (L)	
	AT5G24660	LSU2, protein response to low sulfur 2	response to S deficiency, plant immune responses	Unknown	Y2H (H)	*Arabidopsis* Interactome Mapping Consortium, 2011
					BiFC (L)	Garcia-Molina et al., 2017
					Y2H (L)	
	AT4G33950	OST1, open stomata 1	ABA responses, stomatal closure	nucleus	*in vitro* kinase assay (L)	Wang et al., 2013
FSD3	AT4G28590	ECB1, early chloroplast biogenesis 1, chloroplastic	thioredoxin signaling, plastid gene expression and chloroplast development	plastid, chloroplast stroma, nucleoid	*in vitro* pull-down/WB (L)	Yua et al., 2014
					BiFC (L)	
					Y2H (L)	
CSD1	AT3G14990	DJ-1A, protein DJ-1 homolog A	cytosolic activation of CSD1	cytoplasm, nucleus	BiFC (L)	Xu et al., 2010
					ITC (L)	
	AT1G18080	RACK1A, receptor for activated C kinase 1A	scaffold protein, regulation of signal transduction	cytoplasm, nucleus	SUS (L)	Kundu et al., 2013
CSD1-3	AT1G12520	CCS, copper chaperone for SOD	copper chaperone, CSDs activation	plastid, chloroplast, cytosol	in-gel activity assay (L)	Huang et al., 2012
					Y2H^1^ (H)	*Arabidopsis* Interactome Mapping Consortium, 2011
CSD2	AT1G17870	EGY3, ethylene-dependent gravitropism-deficient and yellow-green 3	chloroplastic ROS homeostasis, retrograde signaling in response to salt stress	plastid, chloroplast membrane	Co-IP/MS (H)	Zhuang et al., 2021
					Co-IP/WB (L)	
					BiFC (L)	
MSD1	AT4G27940	MTM1, manganese tracking factor for MSD 1	MnSOD activation and ion homeostasis	mitochondrion	in-gel activity assay (L)	Hu et al., 2021
					BiFC (L)	
	AT2G46320	MTM2, manganese tracking factor for MSD 2	MnSOD activation and ion homeostasis	mitochondrion	in-gel activity assay (L)	Hu et al., 2021
					BiFC (L)	

ABA, abscisic acid; BiFC, Bimolecular fluorescence complementation assay; Co-IP, Co-immunoprecipitation; FSD, iron superoxide dismutase; FRET, Förster resonance energy transfer; H, high-throughput; ITC, Isothermal titration calorimetry; L, low-throughput; MS, Mass spectrometry; PM, plasma membrane; SUS, Spilt-ubiquitin system; Y2H, Yeast two-hybrid assay; WB, western blotting. ^1^Applies for CSD2 only.

Recently, Co-IP coupled to either MS (Co-IP/MS) or WB (Co-IP/WB) and BiFC assay indicated the interaction of CSD2 with ethylene-dependent gravitropism-deficient and yellow-green 3 (EGY3), a chloroplast membrane-associated ATP-independent metalloprotease (Zhuang et al., 2021). EGY3 is a pseudoprotease showing a high homology with the family of site-2-proteases (S2P), which are involved in proteolytic cleavage of membrane-anchored transcription factors at the plasma membrane. These proteases occur in prokaryotes, mammals and plants, however, EGY3 was identified only in plants with high conservation in the plant kingdom (Adamiec et al., 2017; Adamiec et al., 2020). *EGY3* expression is strongly induced by salt, oxidative stress (Zhuang et al., 2021), high light, and high temperature stress (Adamiec et al., 2022). The stress-induced interaction of CSD2 with M50-like domain of EGY3 stabilizes CSD2 as indicated by decreased abundance of CSD2 under salt stress and high light conditions in *egy3* mutants. The CSD2-EGY3 interaction is important for H_2_O_2_ pool maintenance to ensure chloroplast-nucleus retrograde signaling (Zhuang et al., 2021; Adamiec et al., 2022). The Cu^2+^-dependence of this regulation remains to be revealed.

Several other interactions of CSDs were identified by large-scale OMICs-based analyses (**Table 1**; **Table S1**) including heavy metal-associated isoprenylated plant protein 20 (HIPP20; *Arabidopsis* Interactome Mapping Consortium, 2011), receptor for activated C kinase 1 (RACK1A; Kundu et al., 2013), both for CSD1 only, chitin elicitor receptor kinase 1 (CERK1; Le et al., 2014) for both CSD1 and CSD2, and polyubiquitin 3 (UBQ3; Kim et al., 2013) for CSD1 and CSD3.

Chitin elicitor receptor kinase 1 (CERK1), a pattern recognition receptor responsive to chitin and salt stress (Espinoza et al., 2017), triggers immune responses accompanied with massive ROS production (Erwig et al., 2017). In addition to CSD1 and CSD2, CERK1 putatively interacts with ascorbate peroxidase 1 (APX1), thioredoxin H4 and putative glutaredoxin C4, as detected by a Y2H screen (**Table S1**; Le et al., 2014). Interactions likely take place at the cytoplasmic kinase domain of CERK1, which may possibly synchronize the ROS production with antioxidant defense during plant biotic interactions and salt stress response. The reliability of these findings has to be further verified *in planta*.

Heavy metal-associated isoprenylated plant proteins (HIPPs) are metallochaperones localized in cytosol, endoplasmic reticulum (ER), and nucleus. They are involved in heavy metal homeostasis and detoxification mechanisms, protein folding, removal of non-native proteins by ER-associated degradation, plant-pathogen interactions, and transcriptional responses to cold and drought (de Abreu-Neto et al., 2013; Cowan et al., 2018; Guo et al., 2021). HIPPs typically contain one or two metal binding domains (de Abreu-Neto et al., 2013) present also in CCS structure (N-terminal ATX1-like domain). Based on their structural similarities with SOD-activating CCS proteins (de Abreu-Neto et al., 2013), HIPPs might be considered as putative CSDs activators.

The precise mechanism of Fe^2+^ delivery to the active site of FeSOD was not identified yet. Nevertheless, chloroplastic FeSOD activation is known to be mediated by interaction with chloroplast-localized chaperonin 20 (CPN20), as confirmed by in-gel SOD activity assay, FRET and Y2H (**Table 1**; Kuo et al., 2013b). *In vitro* experiments proved the CPN20 ability to bind Fe^2+^ and incorporate Fe^2+^ to the FSD1-holoenzyme (Kuo et al., 2013a). *CPN20* is involved in the regulation of abscisic acid (ABA) signaling (Zhang et al., 2013b; Guo et al., 2015; Liang et al., 2021) and its overexpression confers increased FSD1 activity in *Arabidopsis*. Additionally, CPN20 likely activates FSD2 and FSD3 as well, since activities of all FSD isoenzymes increased in the presence of CPN20 *in vitro* (Kuo et al., 2013b). The function of this interaction has not been revealed so far. It is quite likely, that it is specific for the regulation of chloroplastic FeSODs with the potential impact on ROS signaling. The mechanism of FSD1 activation in cytosol is still not uncovered and needs further investigation.

FSD2 and FSD3 (exclusively localized in chloroplasts) are components of a plastid-encoded RNA polymerase (PEP) complex involved in chloroplast development and maintenance (Pfannschmidt et al., 2015). Both isoforms possess ROS scavenging activity and might protect nucleoids from oxidative damage (Myouga et al., 2008). Additionally, *in vitro* pull-down assay, BiFC and Y2H showed that FSD3 interacts with early chloroplast biogenesis 1 (ECB1; **Table 1**). ECB1 has, thioredoxin activity and regulates chloroplastic gene expression by affecting the redox status of the PEP complex (Yua et al., 2014). The presence of both FSD2 and FSD3 in the PEP complex and an interaction with ECB1 indicate the possible role of FSD2 and FSD3 in the regulation of chloroplast gene expression. This is supported by the pale green phenotypes of *fsd2* and *fsd3* single mutants and by the arrested chloroplast development in *fsd2/fsd3* double mutant (Myouga et al., 2008). *FSD3* is expressed in two splicing variants (*FSD3* and *FSD3S*) with equal N-termini, but different C-termini, showing different localizations in chloroplasts. FSD3 is localized only to chloroplast nucleoids, while FSD3S was found in entire organelle. Of note, only the *FSD3* splicing variant rescued the *fsd3* phenotype (Lee et al., 2019). Based on these results, phenotypes of both *fsd2* and *fsd3* mutants may suggest an involvement of FSD2 and FSD3 in the regulation of PEP complex, perhaps leading to impaired expression of genes involved in photosynthesis. The question, whether this function is dependent on FSD2 and FSD3 enzymatic activity, still remains unanswered.

FSD2 activity is linked to plant stress responses through the interaction with low sulphur upregulated 1 (LSU1), which was revealed by *in vitro* pull-down assay, Y2H and BiFC (**Table 1**; Garcia-Molina et al., 2017). *LSUs* represent a plant-specific gene family, and recent phylogenetic analysis demonstrated that they belong to a *Spermatophyta*-specific gene family (Uribe et al., 2022). *LSUs* are expressed under various abiotic and biotic stress conditions and coordinate plant immune responses *via* PPIs during simultaneous exposure of plants to abiotic stresses and pathogen attack (Garcia-Molina et al., 2017; Niemiro et al., 2020). LSU1 is localized predominantly in guard cells, and its interaction increased enzymatic activity of FSD2. The LSU1-FSD2 interaction might be implicated in stomatal closure by regulating H_2_O_2_ levels or remodeling gene expression *via* activation of ROS-responsive genes (Garcia-Molina et al., 2017). Interestingly, the guard cell chloroplast relocation of LSU1 upon treatment with *Pseudomonas syringae* virulence effectors hampers FSD2-LSU1 interaction. *LSU1* downregulation is connected with increased disease susceptibility in plants exposed to abiotic stresses while overexpression leads to improved disease resistance (Garcia-Molina et al., 2017). Therefore, the FSD2-LSU1 interaction and following ROS regulation are crucial in combinatorial plant responses to biotic and abiotic stress (Garcia-Molina et al., 2017).

In addition to the aforementioned interacting partners of FSDs, several large-scale, OMICs-based PPI analyses suggested interaction with other proteins having diverse functions (**Table S1**). These included chloroplast import (translocase of chloroplast 132 (TOC132); Dutta et al., 2014) and protein degradation (UBQ3; Igawa et al., 2009) for FSD1. This SOD isoform might be also a component of a G-protein complex, since it was shown to interact with guanine nucleotide-binding protein subunit beta (AGB1), guanine nucleotide-binding protein alpha-1 subunit (GPA1), and an effector of its beta subunit called acireductone dioxygenase 2 (ARD2; Friedman et al., 2011; Klopffleisch et al., 2011). FSD2 has been suggested to interact with cysteine/histidine-rich c1 domain family protein, btb/poz domain protein, ubiquitin-conjugating enzyme 27 (UBC27; *Arabidopsis* Interactome Mapping Consortium, 2011), btb/poz and math domain-containing protein 3 (BPM3; Altmann et al., 2020), open stomata 1 (OST1; Wang et al., 2013), and UBQ3 (Kim et al., 2013), which are localized in cytosol. The suggested interacting proteins are not localized or functionally connected to chloroplast. Therefore, it is unlikely that these proteins interact with the mature FSD2 protein. Nevertheless, the interaction of above-mentioned proteins with the nascent FSD2 protein cannot be ruled out. Teosinte branched 1/cycloidea/pcf (TCPs) are highly conserved plant-specific transcription factors with preferential localization in cytosol and nucleus (Martín-Trillo and Cubas, 2010). It was suggested by Y2H approach, that FSD2 interacts with TCP13 and TCP15, both possessing chloroplast-targeting sequence (**Table S1**; Baba et al., 2001; Wagner and Pfannschmidt, 2006; Martín-Trillo and Cubas, 2010). TCP13 is involved in the regulation of gene expression in chloroplasts (Baba et al., 2001) and plays important roles in leaf and root growth during abiotic stress (Urano et al., 2022). Similar roles have been suggested for TCP15 (Wagner and Pfannschmidt, 2006; Martín-Trillo and Cubas, 2010). FSD2-TCP interaction implies additional possible mechanism of FSD2-mediated regulation of chloroplastic genes expression.

The first mechanism of mitochondrial MnSOD activation was described for yeast SOD2, which is activated by interaction with manganese trafficking factor for mitochondrial SOD2 (MTM1; Luk et al., 2003). Recently, the functional homologs of yeast MTM1 (yMTM1) were identified in *Arabidopsis*. AtMTM1 and AtMTM2 are mitochondrial carriers with conserved transmembrane sequences interacting with MSD1, as found by both in-gel SOD activity assay and BiFC (**Table 1**). AtMTM1 and AtMTM2 can activate yeast SOD2 in *yMTM1*-mutant cells, which points to their high homology with yMTM1 (Hu et al., 2021). Additionally, both MTM isoforms are involved in Mn^2+^ and Fe^2+^ homeostasis, regulation of flowering time and root elongation (Hu et al., 2021; Hu and Jinn, 2022), the latter function being also associated to MSD1 (Morgan et al., 2008).

High-throughput CF-MS analysis identified several important metabolic proteins as potential MSD1 interactors (**Table S1**; McWhite et al., 2020). Notably, interactions with enzymes of tricarboxylic acid cycle, such as mitochondrial malate dehydrogenases (mMDHs) and succinate–CoA ligase subunits (SCS-alpha-1/2 and SCS-beta), may be related to the repression of tricarboxylic acid cycle flux in isolated mitochondria in *MSD1* RNAi lines (Morgan et al., 2008).

Taken together, SODs may regulate gene expression *via* PPIs, and are essential constituents of the ROS signaling pathways, at least partially by affecting subcellular pools of H_2_O_2_.

## Interaction partners of catalases

Catalases (CATs) are iron-containing homo-tetrameric metalloenzymes that catalyze the decomposition of H_2_O_2_ to water and oxygen and play a central role in the regulation of H_2_O_2_-signaling during plant responses to abiotic and biotic stresses (Yuan et al., 2017; Su et al., 2018; Yang et al., 2019a). Three main CAT families evolved during the evolution, including heme-typical CATs, catalase-peroxidases, and minor manganese CATs. Typical CATs represent a most abundant group occurring in most living organisms, including eubacteria, archaebacteria, protista, fungi, animals and plants (Zamocky et al., 2008; Pan et al., 2022). Although plant CATs are recognized as peroxisomal proteins, the presence of CATs has been also detected in nucleus (Al-Hajaya et al., 2022), cytoplasm, mitochondria and chloroplasts (Mullen et al., 1997). Three CAT isoforms (CAT1, CAT2, and CAT3) documented in *Arabidopsis* have diverse developmental and stress-related functions (Palma et al., 2020). Expression pattern of *CATs* is tightly regulated by numerous transcription factors (Wang and Chu, 2020; Chu et al., 2021; Dvořák et al., 2021b; Song et al., 2021). PTMs, such as phosphorylation, glycation, Tyr-nitration, acetylation, S-nitrosation regulate CATs functions (Zou et al., 2015; Palma et al., 2020; Zhang et al., 2020). Notably, CATs are frequently bound and inhibited by pathogen effectors leading to pathogen spread and mitigation of host immunity (Murota et al., 2017; Sun et al., 2017; Jiao et al., 2021; Yuan et al., 2021).

CATs interact with numerous proteins of diverse functions. CATs peroxisomal targeting is mediated by their interaction with peroxisomal targeting signal 1 receptor (PEX5; **Table 2**; Oshima et al., 2008; Freitas et al., 2011). CATs are subsequently released by PEX14, allowing CAT tetramerisation (Freitas et al., 2011). A recent study revealed the importance of a C-terminal heme binding domain of CAT for its peroxisomal targeting (Fujikawa et al., 2019).

**Table 2:** Interaction partners of *Arabidopsis* catalases found by low-throughput methods with their respective function and localization.

Protein of interest	Accession	Interactor name	Function	Localization	Method of detection	Reference
CAT1/2	AT5G56290	PEX5, peroxisome biogenesis protein 5	peroxisome protein import, receptor for peroxisomal-targeting signal one (PTS1)	cytoplasm, peroxisome membrane	Y2H (L)	Oshima et al., 2008
CAT1/2/3	AT3G54360	NCA1, no catalase activity 1	holdase chaperone activity towards catalase	cytoplasm, nucleus	BiFC^1^ (L)	Li et al., 2015
					Co-IP/WB (H)	
					holdase chaperone assay^1^ (L)	
					*in vitro* pull-down/WB^1^ (L)	
	AT4G20380	LSD1, lesion simulating disease 1	stress-induced cell death, regulator of CSD1 and CSD2	cytoplasm, nucleus	Co-IP/WB (L)	Li et al., 2013
					Y2H (L)	
	AT4G09320	NDPK1, nucleoside diphosphate kinase 1	NTP synthesis, oxidative stress response	peroxisome, nucleus, cytoplasm	Y2H (L) non-denaturing 2D-PAGE	Fukamatsu et al., 2003
CAT2	AT5G12020	HSP17.6II, 17.6 kDa class II heat shock protein	catalase chaperone	cytoplasm, peroxisome	IP/MS (H)	Li et al., 2017b
					IP/WB (L)	
					CAT activity assay (L)	
					BiFC (L)	
	AT4G30920	LAP2, leucyl aminopeptidase 2	senescence, stress response and amino acid turnover	cytoplasm, chloroplast	Y2H (L)	Zhang et al., 2021
					BiFC (L)	
					IP/WB (L)	
					SLC (L)	
CAT2/3	AT5G35410	SOS2, salt overly sensitive 2	control of intracellular Na^+^ and K^+^ homeostasis	cytoplasm, nucleus	Y2H (L)	Verslues et al., 2007
					TAP-MS (H)	
	AT4G33050	IQM1, IQ-motif containing protein 1	Calcium-independent calmodulin-binding protein, JA biosynthesis, defense against the necrotrophic pathogen *Botrytis cinerea*	nucleus, cytoplasm, peroxisome	Y2H (L)	Lv et al., 2019
					IP/WB (L)	
					BiFC(L)	
					CAT activity assay^1^ (L)	
	AT4G19200	GPRP3, Glycine- and Proline-Rich Protein	plant growth, environmental adaptation	nucleus, cytoplasm	Y2H (L)	Liu et al., 2020
					BiFC (L)	
CAT3	AT5G19450	CDPK19, calcium-dependent protein kinase 8	calcium signaling	plasma membrane	BiFC (L)	Zou et al., 2015
					Co-IP/WB (L)	
					Y2H (L)	
					*in vitro* kinase assay (L)	
					SLC (L)	

2D-PAGE, two-dimensional gel electrophoresis; BiFC, Bimolecular fluorescence complementation assay; Co-IP, Co-immunoprecipitation; H, high-throughput; IP, immunopurification; L, low-throughput; MS, Mass spectrometry; SLC, spilt-luciferase complementation assay; TAP, Tandem affinity tag purification; WB, western blotting; Y2H, Yeast two-hybrid assay. ^1^Applied for CAT2 only.

*Arabidopsis* CATs are activated by no catalase activity 1 (NCA1), a cytosolic chaperone protein. Interactions between CATs and NCA1 were uncovered by Co-IP/WB and for CAT2 further validated by BiFC, *in vitro* pull-down assay coupled to WB and holdase chaperone assay (**Table 2**; Li et al., 2015). CAT2 interacts explicitly with the C-terminal tetratricopeptide repeat-like helical domain, while the N-terminal RING-finger domain of NCA1, containing zinc ion, is required for its activation. CAT2 activity increased 10-fold after interaction with NCA1 *in vitro*. In addition, *nca1* mutants exhibit increased sensitivity to salt, cold and oxidative stress (Li et al., 2015), suggesting that NCA1-CAT interaction might be important for ROS homeostasis. Moreover, reverse genetic studies indicated that NCA1-CAT interaction may regulate immunity-triggered autophagy (Hackenberg et al., 2013). Based on the known chaperone function of NCA1, it is likely that NCA1-CAT pair may mediate optimal folding of nascent CAT in the cytosol. The *NCA1* gene is present solely in plants and in some species such as *Oryza sativa* or *Nicotiana tabacum* it exists in two copies (Liu et al., 2019). Interestingly, both gene products retained the ability to bind CAT (Liu et al., 2019). These results point to the evolutionary conservation of CAT-NCA1 interaction.

Another CAT activation mechanism is based on CAT2 interaction with small heat shock protein Hsp17.6CII, a peroxisome-localized catalase chaperone, as examined by BiFC, Co-IP/MS and Co-IP/WB (**Table 2**; Li et al., 2017b). Small heat shock proteins (sHSPs) are ubiquitous chaperones involved in stress responses of prokaryotic and eukaryotic cells (reviewed in Waters, 2013; Haslbeck and Vierling, 2015). Overexpression of *Hsp17.6CII* in *nca1* mutant did not lead to increased stress resistance and CAT activity, indicating that Hsp17.6CII activates CAT2 in an NCA1-dependent manner. Moreover, overexpression of *Hsp17.6CII* in wild type led to increased CAT activity and transgenic lines exhibited increased tolerance to abiotic stresses (Li et al., 2017b). Finally, cassava chaperone MeHSP90.9 has been identified by Y2H, BiFC and pull-down assay combined with WB as a positive regulator of MeCAT1 activity which plays an important regulatory role during drought stress resistance (Wei et al., 2020). These results highlight HSPs as another important part of CATs regulation, mainly in stress conditions.

Multiple low and high-throughput examinations proved that all three CATs interact with zinc finger protein lesion simulating disease 1 (LSD1; **Table 2**) which is localized in the nucleus and cytoplasm. LSDs are present exclusively in *Viridiplantae* with highly conserved structural domains (Li et al., 2013; Cabreira et al., 2015). LSD1 is a scaffold protein and transcriptional regulator during biotic and abiotic stress responses of plants. Moreover, it plays an essential role as a negative regulator of programmed cell death (PCD) and its numerous PPIs are redox-dependent (Li et al., 2013; Czarnocka et al., 2017). The interaction between the LSD1 and CATs required the presence of all three LSD1-like zinc finger motifs and is involved in light-dependent runaway cell death and hypersensitive response cell death regulation in *Arabidopsis* (Li et al., 2013). Based on the high conservation of LSD1 in plants it is likely that this regulation mechanism also occurs in other plant species (Cabreira et al., 2015), however, no other experimental evidence has been reported to this date. Nevertheless, a recent study reported that interaction of cassava MeLSD3 with MeAPX2 promoted its activity and increased antioxidant efficiency (Zeng et al., 2022). Therefore, LSDs may ensure the coordination of antioxidant defense during cell death.

CATs involvement in antioxidant defense might be mediated by the interaction with nucleoside diphosphate kinase (NDPK), a housekeeping enzyme which catalyzes interconversion of nucleoside diphosphates and triphosphates (Dorion and Rivoal, 2018). This interaction, important for development, stress and light responses, was identified in *Arabidopsis* by Y2H and non-denaturing two-dimensional gel electrophoresis (**Table 2**; Fukamatsu et al., 2003). It was confirmed in fungus *Neurospora crassa* (Yoshida et al., 2006; Wang et al., 2007; Lee et al., 2009) and *Pisum sativum* (Haque et al., 2010), showing high degree of conservation. NDPK2 and CAT2/3 form a complex with salt overly sensitive 2 (SOS2), known as a class 3 sucrose-nonfermenting 1-related kinase as revealed by Y2H and TAP-MS analyses (**Table 2**; Verslues et al., 2007). This complex is involved in crosstalk between salt stress response and ROS signaling (Verslues et al., 2007). NDPK is known to mediate the expression of antioxidant enzymes such as SODs, peroxidases, CAT, APX, thioredoxin reductase and peroxiredoxin in plants (Yang et al., 2003; Tang et al., 2008; Zhang et al., 2017). Thus, it might be hypothesized that NDPKs govern antioxidants also by other mechanisms, for example, by interaction with mitogen-activated protein kinases MPK3 and MPK6 (Moon et al., 2003), which may initiate expression of antioxidant enzymes (Dvořák et al., 2021b).

CAT1 might also bind to two proteins, which interact with target of rapamycin (TOR) kinase suggested by large scale TAP-MS (**Table S2**; Van Leene et al., 2019). Both regulatory-associated protein of mTOR 1B (RAPTOR 1B) and lethal with Sec thirteen 8/G protein β subunit-like (LST8/GβL), bear a WD-40 repeat motif, indicating possible affinity of CAT to this domain conferring PPIs or protein-DNA interactions. TOR kinase integrates nutrient-, energy- and stress-related cues with growth and metabolic outputs while above mentioned interactors are supposed to control target specificity and the stability of TOR complexes (Rexin et al., 2015).

CAT2 and CAT3 interact with the calmodulin-binding protein IQ-motif containing protein 1 (IQM1) as confirmed by Y2H, Co-IP/WB, BiFC and CAT activity assays (**Table 2**). IQM1 modulates CAT2 activity which indirectly stimulates the JA biosynthetic enzymes such as acyl-CoA oxidases in response to *Botrytis cinerea* infection (Lv et al., 2019). On the other hand, IQM1 negatively regulates plant ABA responses and stomatal movement by affecting ROS levels (Zhou et al., 2012). Proteins homologous to IQM1 were found in rice and most OsIQM members are responsive to ABA, polyethylene glycol and salt, implying their involvement in stress responses (Fan et al., 2021).

CAT2 is stabilized during salt and drought stresses by an interaction with leucine aminopeptidase 2 (LAP2), which was shown by several independent methods such as Y2H, BiFC, IP/WB, and spilt-luciferase complementation assay (**Table 2**). It was found that this interaction is important also for stability and increased activity of LAP2 in order to increase gamma aminobutyric acid (GABA) content as a response to salt and osmotic stress (Zhang et al., 2021). *AtLAP2* is homologous to tomato *LAP-A* and *LAP-N*, which act as molecular chaperones and facilitate the stability of proteins during stresses (Scranton et al., 2012), implying that tomato LAPs share the CAT stabilization ability with LAPs of *Arabidopsis*.

AP-based interaction studies identified two members of 14-3-3 protein family (**Table S2**), 14-3-3-like protein GF14 omega (GRF2; Chang et al., 2009) and 14-3-3-like protein GF14 psi (GRF3; Shin et al., 2011), as another putative interaction partners of CAT2 and CAT3, respectively. 14-3-3 proteins bind multiple metabolic proteins and proteins involved in signaling in order to regulate plant development and biotic and abiotic stress responses (Roberts et al., 2002). GRF2 is involved in responses to salt stress by regulating H^+^-ATPase activity that provides proton gradient for Na^+^/H^+^ antiporter SOS1, a transport protein regulated by SOS2, another interaction partner of CATs (Verslues et al., 2007; Yang et al., 2019b). This points to the close crosstalk of CATs with the SOS pathway.

High-throughput assays indicated several nuclear CAT interactors (**Table S2**), which may shed light on the recently discovered nuclear targeting of CAT (Al-Hajaya et al., 2022). CAT isoforms might be associated with the nuclear pore complex (NPC) as indicated by the possible interaction of all three CATs with nuclear pore complex protein 43 (NUP43; also known as nucleoporin). It belongs to WD-40 repeat protein family and plays a role in nucleocytoplasmic transport (Tamura et al., 2010). Interestingly, CAT3 binds to Nup93/Nic96 nucleoporin interacting component-containing protein, a component of the NPC (Li and Gu, 2020) and with RNA export factor 1 (RAE1), a protein binding to NUP98, involved in mRNA export and cell division (Meier and Brkljacic, 2009). These data point to possible role of CAT in nucleocytoplasmic transport.

Other nuclear CAT interactors involve histone H3 acetyltransferase (increased DNA methylation 1 (IDM1); applied for CAT3) and defective in meristem silencing 3 (DMS3), both involved in epigenetic modifications (Kanno et al., 2008).

## PPIs of ascorbate-glutathione cycle enzymes

APX is a central enzyme of the ascorbate-glutathione cycle, a major pathway of chloroplastic H_2_O_2_ elimination and a redox balancing mechanism in adverse environmental conditions. Within this cycle, APX decomposes H_2_O_2_ using ascorbate as an electron donor. Ascorbate is recycled by either monodehydroascorbate reductase (MDAR) or dehydroascorbate reductase (DHAR; Bartoli et al., 2017).

APXs are responsive to external (abiotic and biotic stresses) and internal (phytohormones) stimuli. This enzyme encoded by several compartmentalized isoforms is regulated on transcriptional and posttranslational level (Maruta et al., 2016). The expression of cytosolic *APX* strongly depends on the redox state of plastoquinone pool, acting as a sensor of light intensity (Karpinski et al., 1997). APXs are targeted by redox buffering proteins such as TRXs (Gelhaye et al., 2006; Pérez-Pérez et al., 2017) or nucleoredoxin (Kneeshaw et al., 2017). In addition, APX functions are fine-tuned by PPIs. APXs are bound by chloroplast-targeted APX-related (APX-R) proteins, which are structurally related to APXs (Lazzarotto et al., 2011) and operate in ascorbate-independent manner (Lazzarotto et al., 2021). The functional relevance of this interaction remains to be elucidated.

An intricate interaction was detected between cytosolic NtcAPX and cell division cycle 48 (NtCDC48) in tobacco by high-throughput Co-IP/MS (Rosnoblet et al., 2017), FRET-FLIM and pull-down assays (Bègue et al., 2019). A chaperone-like CDC48, a protein evolutionary highly conserved in eukaryotes and archaea, regulates the proteasome-mediated degradation and autophagy during development as well as abiotic and biotic stress responses (Park et al., 2008; Stolz et al., 2011; Bègue et al., 2017; Rosnoblet et al., 2021). It undergoes oxidative modifications and can segregate and remodel its interacting partners from complexes or cellular structures (Rosnoblet et al., 2021). Some interactions of CDC48 described in archaea and eukaryotes are also evolutionarily conserved (Barthelme and Sauer, 2013). *NtCDC48* negatively affects *NtcAPX*, since its overexpression suppresses *NtcAPX* expression, abundance, and activity during elicitation with cryptogein and heat shock. Thus, NtCDC48-*NtcAPX* interaction contributes to cAPX protein turnover (Bègue et al., 2019). Furthermore, according to high-throughput Co-IP/MS interaction studies NtCDC48 is supposed to interact with NtMnSOD and NtCAT1 (Rosnoblet et al., 2017), pointing to its broader impact on antioxidant defense. Interestingly, AtCDC48A controls the degradation of intrachloroplastic proteins during oxidative stress and is vital for methyl viologen-induced oxidative stress tolerance (Li et al., 2022). Indeed, abundances of cytosolic, stromal and thylakoid APX are hypersensitive to highly oxidative conditions induced by prolonged exposure to methyl viologen (Melicher et al., 2022). Thus, CDC48 may govern APX, SOD and CAT degradation to modulate plant antioxidant defense and may play a key role as a regulator of the cellular redox status.

Using Co-IP/MS, APX1 was found to interact with plasma membrane-localized aquaporins PIP2A, PIP1B and ammonium transporter 1.3 (AMT1;3; Bellati et al., 2016; **Table S3**). In addition, high throughput Y2H study indicated an interaction with CERK1, which also resides in plasma membrane (**Table S3**; Le et al., 2014). Although the false positivity of these identifications cannot be ruled out, they may interact with diverse cytosolic proteins *via* their cytosolic loops, as it was described for PIPs (Roche and Törnroth-Horsefield, 2017). APX1 co-purified with nucleolar A2-type cyclin CYCA2.3 in a TAP-MS experiment (**Table S3**; Boudolf et al., 2009). However, due to different localizations, the above-mentioned interaction remains to be validated by additional experiments.

Low-throughput Co-IP and Y2H assays revealed, that two peroxisomal membrane-bound APX isoforms APX3 and APX5 interact with cytosol- and nucleus-localized ankyrin repeat-containing protein 2A (AKR2A; **Table 3**; Shen et al., 2010). AKR2A interacts *via* N-terminal PEST (named after Pro-Glu-Ser-Thr) domain with the C-terminal transmembrane domain of nascent APXs to maintain their stability and to prevent protein aggregation (Shen et al., 2010). Indeed, AKR2A may bind and stabilize nascent proteins targeted to diverse organelles, such as chloroplast outer envelope membrane (OEM) proteins, contributing to chloroplast biogenesis and protein trafficking (Bae et al., 2008; Kim et al., 2015). *AKR2A* overexpression leads to upregulation of enzymes involved in H_2_O_2_ signaling, including APX1 and secretory peroxidase isoforms (Hu et al., 2020), indicating that AKR2A may have a broader impact on antioxidant defense. Of note, AKR2A undergoes oxidative modifications on Cys259 (S-nitrosylation and S-sulfenylation) and on Cys266 (S-sulfenylation; Hu et al., 2015; Huang et al., 2019), enabling possible feedback regulation of APXs and rapid responsivity to fluctuating cellular redox homeostasis. Plant AKR2A comprises four conserved amino acid regions, including the above-mentioned PEST sequence, two central domains, and a C-terminal ankyrin repeat domain (ARD). Interestingly, the PEST sequence exists only in land plants, while the C-terminal ARD domain has the highest conservation across prokaryotes, eukaryotes, green algae and land plants (Kim et al., 2014). This suggests that APX3-AKR2A interaction occurs solely in land plants.

**Table 3:** Interaction partners of *Arabidopsis* ascorbate-glutathione cycle enzymes found by low-throughput methods with their respective function and localization.

Protein of interest	Accession	Interactor name	Function	Localization	Method of detection	Reference
APX3	AT4G23650	CPK3, calcium-dependent protein kinase 3	calcium signaling, stomatal closure	cytoplasm, nucleus	FRET (L)	Berendzen et al., 2012
					BiFC (L)	
	AT5G10450	GRF6, 14-3-3-like protein GF14 lambda	cold stress and brassinosteroid signaling	cytoplasm, nucleus, plasma membrane	Y2H (L)	Zhang et al., 1997
APX3/5	AT4G35450	AKR2A, ankyrin repeat domain-containing protein 2A	molecular chaperone, protein targeting to chloroplasts, ROS regulation	cytoplasm, nucleus, plastid, chloroplast outer membrane	Co-IP/WB (L)	Yan et al., 2002
					Co-IP/WB (L)	Shen et al., 2010
					Y2H (L)	Shen et al., 2010
APX5	AT2G17390	AKR2B, ankyrin repeat domain-containing protein 2B	molecular chaperone, protein targeting to chloroplasts	cytoplasm, nucleus, plastid chloroplast outer membrane	Y2H (L)	Shen et al., 2010
GR2	AT2G19080	MTX1, mitochondrial outer membrane import complex protein METAXIN	transport proteins into the mitochondrion	mitochondrion outer and inner membrane	Y2H (L)	Lister et al., 2007
	AT5G09420	TOM64, outer envelope protein 64, mitochondrial	chaperone receptor, mitochondrial protein transport	mitochondrion outer membrane	Y2H (L)	Lister et al., 2007
	AT1G27390	TOM20-2, mitochondrial import receptor subunit	transit peptide receptor, mitochondrial protein transport	mitochondrion outer membrane	Y2H (L)	Lister et al., 2007
	AT3G27080	TOM20-3, mitochondrial import receptor subunit	transit peptide receptor, mitochondrial protein transport	mitochondrion outer membrane	Y2H (L)	Lister et al., 2007
	AT5G40930	TOM20-4, mitochondrial import receptor subunit	transit peptide receptor, mitochondrial protein transport	mitochondrion outer membrane	Y2H (L)	Lister et al., 2007

BiFC, Bimolecular fluorescence complementation asssay; Co-IP, Co-immunoprecipitation; FRET –Förster resonance energy transfer; L, low-throughput; Y2H, Yeast two-hybrid assay.

According to low-throughput Y2H and Co-IP/WB analyses, both AKR2A and APX3 interact with a 14-3-3-like protein GF14 lambda (GRF6; **Table 3**; Zhang et al., 1997; Yan et al., 2002). Although GRF6 is not required for ANKR2A-APX3 interaction, it is proposed that this complex modulates the APX3-mediated antioxidant defense during stress responses.

Considering that interaction of ANKR2A-APX3 occurs in the cytosol, the positive effect of ANKR2A on APX3 stability might allow APX3 to interact with other cytosolic proteins, such as Ca^2+^-dependent protein kinase 3 (CPK3; Berendzen et al., 2012), thus linking APX3 with Ca^2+^ signaling. The function of this interaction, examined by FRET-FLIM and BiFC assay combined with flow cytometry (Berendzen et al., 2012), was not described in detail. CPKs were identified in plants and protozoans, but not in animal kingdom or yeast (Harmon et al., 2000), and are involved in various signaling pathways during biotic and abiotic stress responses. In *Arabidopsis*, CPK3 modulates salt stress response by phosphorylation of vacuolar two-pore K^+^ channel 1 (AtTPK1; Latz et al., 2013). Notably, AtTPK1 interacts with GRF6 *via* its pSer42 residue (Latz et al., 2007), which is phosphorylated by CPK3. As noted above, CPK3 and GRF6 are verified APX3 interaction partners (**Table 3**), therefore APX3, GRF6 and CPK3 may form a complex involved in the regulation of salt stress response.

The interactions of stromal sAPX and thylakoid tAPX have not been resolved so far.

MDARs, enzymes using NADH and NADPH as electron donors compartmentalize into cytosol, chloroplasts, mitochondria, and peroxisomes (Tanaka et al., 2021) and are crucial for plant abiotic and biotic stress responses (Li et al., 2010; Yeh et al., 2019). They are regulated on transcriptional and posttranscriptional levels (Feng et al., 2014). Nevertheless, the mechanism of their activation is only scarcely known. MDAR1 and MDAR2 are subjected to phosphorylation and nitration in response to diverse stimuli (Begara-Morales et al., 2015; Dvořák et al., 2021b) and they are modulated also by thioredoxins (Vanacker et al., 2018).

As shown by a large-scale study applying CF-MS (**Table S3**; McWhite et al., 2020), MDAR1/2/3 and 6 belong to a multiprotein complex comprised mainly of metabolic proteins. These include two isoforms of mitochondrial malate dehydrogenase, essential for central metabolism, photorespiration and redox homeostasis between organelles (Sun et al., 2019). In addition, mitochondrial succinyl-CoA synthetase activity is present as well. Transaldolase 2 (TRA2), regulating the balance of metabolites in the pentose-phosphate pathway, and proteins involved in carbohydrate partitioning, sucrose synthesis, and cell wall biogenesis are also components of this complex, namely, two isoforms of phosphoglucomutase 2 and 3 (PGM2 and PGM3), cytosolic and plastidial isoform of triosephosphate isomerase (TPI and TIM, respectively), and UTP-glucose-1-phosphate uridylyltransferase 1 and 2 (UGP1 and UGP2). Thus, MDARs might stand for unique enzymes adapting the primary metabolism to redox homeostasis perceived by ascorbate-glutathione pathway.

*Arabidopsis* has two cytosolic (DHAR1 and DHAR2) and one chloroplastic (DHAR3) DHAR isoforms. They are highly responsive to a wide range of stimuli and their overexpression confers plant resistance to abiotic stresses (Chen and Gallie, 2006; Noshi et al., 2017). Their expression and activity are linked to MAPK signaling, Ca^2+^ levels, and expression of transcription factors (Dvořák et al., 2021b). However, direct regulation of DHARs by TFs was not experimentally proved so far. In addition, DHARs undergo S-glutathionylation (Dixon et al., 2005) and have been identified as thioredoxin targets (Hägglund et al., 2008).

DHAR1 interactors have been found mainly by large-scale study exploiting a mating-based split-ubiquitin system accommodated for membrane protein interactions with other membrane or cytosolic proteins (**Table S3**; Jones et al., 2014). This approach identified 46 potential DHAR1 membrane-localized interacting partners, mainly involving receptors and transporters. DHAR1 may interact with 5 members of multidrug and toxic compound extrusion (MATE) protein family involved in plant resistance to multiple cytotoxic compounds, including antibiotics and heavy metals. They are also capable of transporting flavonoids or anthocyanins and have developmental functions (Remy and Duque, 2014; Suzuki et al., 2015; Miyauchi et al., 2017). Two of these interactions (DTX20 and DTX14) were verified by *in planta* by GFP split assay (Jones et al., 2014), increasing the reliability of the interaction identification.

DHAR1 may also interact with nitrate transporter 1.12 (NTR1.12), glucose transporter 1 (GLT1; both interactions proved *in planta)*, proteins transporting phosphoenolpyruvate (pep)/phosphate (phosphoenolpyruvate (pep)/phosphate translocator 2; (PPT2)), H^+^ or Na^+^ (cation/H^+^ antiporter 27; (CHX27)), GABA (putative GABA transporter 2 (GAT2)), L-cystine (cystinosin homolog), sucrose (bidirectional sugar transporter SWEET9) and UDP-xylose (UDP-xylose transporter 1 (UXT1); **Table S3**; Jones et al., 2014). This implies possible novel functions of DHAR1, linking redox homeostasis to membrane integrity ensuring transmembrane transport of different compounds. Cytosolic DHAR1, DHAR2 and DHAR3 interactions have not been studied so far.

Glutathione reductases (GRs) are catalyzing the NADPH dependent recovery of reduced glutathione consumed by DHAR activity. Thus, they substantially contribute to glutathione recycling. *Arabidopsis* GR isoforms are localized in the cytosol, peroxisomes (GR1), chloroplasts and mitochondria (GR2; Gill et al., 2013). They are important for ROS regulation under diverse stimuli (Müller-Schüssele et al., 2020). According to a large-scale study exploiting CF-MS (**Table S3**; McWhite et al., 2020), GR1 and GR2 are members of a complex containing two isoforms of protein disulfide isomerase-like proteins PDIL1-1 and PDIL1-2, proteins involved in oxidative protein folding (Fan et al., 2022) and two chloroplastic transketolase isoforms TKL1 and TKL2 involved in carbon metabolism (Rocha et al., 2014). The cytosolic and membrane anchored aminopeptidase M1 (APM1), a potential interactor of GR1 and GR2, is involved in auxin transport as knockout mutant in *APM1* shows irregular cell divisions during embryogenesis (Peer et al., 2009). Other proteins represent enzymes involved in primary (Dihydroxy-acid dehydratase (DHAD), 2-hydroxyacyl-CoA lyase (HACL), pyridoxine/pyridoxamine 5’-phosphate oxidase 1 (PPOX1), sulfoquinovosyldiacylglycerol 1 (SQD1)) or secondary metabolism (chorismate synthase (EMB1144)). Additionally, GR2 interacts with components of mitochondrial protein transport (**Table 3**) including TOM20 mitochondrial import receptor subunits TOM20-2/3 and 4, mitochondrial outer envelope protein 64 (TOM64) and mitochondrial outer membrane import complex protein METAXIN (**Table 3**; Lister et al., 2007), which is in accordance to GR2 mitochondrial localization.

## RACK1 as a putative scaffold protein for antioxidant defense

**Figure 1:**
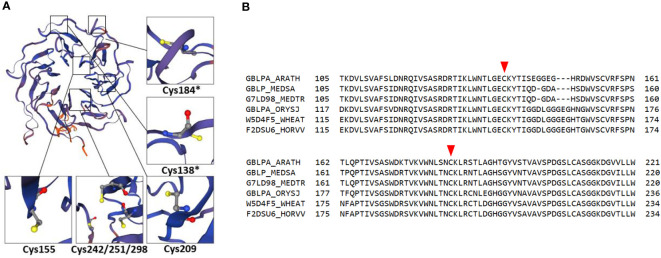
Redox-active cysteines in RACK1A amino acid sequence. **(A)** RACK1A tertiary structure model with detail view of cysteine residues. Asterisks indicate redox-active cysteine residues with high surface accessibility. The model is based on maltose-binding protein fused with RACK1A from *Arabidopsis* and was built using Expasy SWISS-MODEL software (Bienert et al., 2017; Waterhouse et al., 2018). **(B)** Alignment of RACK1A amino acid sequence ranging from 105 to 221 amino acids from *Arabidopsis* with orthologous proteins in different plant species. Orthologous proteins from *Oryza sativa* (GBLPA_ORYSJ), *Hordeum vulgare* (F2DSU6_HORVV), *Triticum aestivum* (W5D4F5_WHEAT), *Medicago sativa* (GBLP_MEDSA), and *Medicago truncatula* (G7LD98_MEDTR) are aligned to *Arabidopsis* RACK1A (GBLPA_ARATH) protein sequence. Red arrowheads indicate redox-active conserved cysteines.

RACK1 comprises seven repetitive WD-40 motifs with β-propeller structure that confers PPI capabilities (Adams et al., 2011; **Figure 1A**). It is conserved in all eukaryotic organisms with high DNA sequence identity and exhibits high sequence and structural conservation among diverse plant species (Iwasaki et al., 1995; McKhann et al., 1997; Chen et al., 2006a; **Figure 1B**). It is involved in plant developmental processes as well as responses to biotic and abiotic stresses (Zhang et al., 2013a; Islas-Flores et al., 2015). In *Arabidopsis*, three RACK1 isoforms are encoded by *RACK1A*, *RACK1B* and *RACK1C* genes (Chen et al., 2006a), while there are two isoforms in rice (*OsRACK1A* and *OsRACK1B*; Nakashima et al., 2008) and one in alfalfa (*Msgb1*; McKhann et al., 1997). It is localized to cytosol and nucleus where it may interact with various proteins (Speth et al., 2013). Currently, BioGRID, a database of protein, genetic and chemical interactions (Oughtred et al., 2021) reports 295 protein interactors for *Arabidopsis RACK1A* and at least 17 of them were validated by more than one PPI method so far.

In plants, *RACK1* is a crucial regulator of ABA responses during seed germination, root growth and cotyledon greening (Guo et al., 2019). *RACK1* affects ABA signaling through the interaction with eukaryotic translation initiation factors (Guo et al., 2011) or proteins involved in miRNA biogenesis (Speth et al., 2013). Genetic modification of *RACK1A* expression is associated with alteration of genes involved in ABA biosynthesis (Zhang et al., 2018). In addition, RACK1 serves as a scaffold for MAPK cascade in response to bacterial protease IV and ArgC in *Arabidopsis* (Cheng et al., 2015).

A growing evidence shows that RACK1 significantly contributes to ROS homeostasis by regulating either ROS generation or scavenging. RACK1 interacts with NADPH oxidase, a plasma-membrane localized superoxide anion-generating enzyme implicated in plant immune responses, abiotic stress tolerance and development (Sagi and Fluhr, 2006). OsRACK1A activates NADPH oxidase encoded by *RbohB* gene by recruiting its activator Rac1 to Rac1-immune complex as found by low-throughput Y2H, split-ubiquitin and Co-IP assays, and leads to increased ROS production (Nakashima et al., 2008). ROS generation mediated by RACK1-RBOH interaction is important for ABA responses as well (Nakashima et al., 2008; Zhang et al., 2014; Li et al., 2017a).

In addition to NADPH oxidases, genetic modification of RACK1 often causes alterations of enzymes involved in ROS decomposition. For example, downregulation of *OsRACK1A* enhanced SOD activity, decreased lipid peroxidation and increased polyethylene glycol-mediated drought stress tolerance in rice (Li et al., 2009). The impact of RACK1 on antioxidants might be broader, because soybean *GmRACK1*-RNAi lines displayed higher *CAT*, *SOD*, *APX* and *GST* transcript levels and SOD, POD and CAT activities during drought stress. On the other hand, *GmRACK1* overexpressing lines exhibited reduced SOD, POD and CAT activities, which correlated with increased ROS levels (Li et al., 2018).

As revealed by high throughput studies, one possible mechanism how RACK1 may modulate antioxidant enzymes is through PPIs (**Table 4**). Antioxidant enzymes, including those involved in detoxification of superoxide and H_2_O_2_ were identified as putative RACK1-interacting partners (**Table 4**). Cytoplasmic and nuclear localization of RACK1A suggests that this scaffold protein should be effective and functionally important mainly in these subcellular compartments. It might be expected, that interactions of RACK1s with antioxidant enzymes will modulate their activity or enable/prevent interaction with other regulatory factors.

**Table 4:** Known protein-protein interactions of receptor for activated C kinase 1 (RACK1) with antioxidant enzymes.

Accession	Interactor name	Function	Localization	Method of detection	Throughput	Reference
AT1G07890	APX1, L-ascorbate peroxidase 1, cytosolic	hydrogen peroxide detoxification	cytoplasm	Y2H	High	Guo et al., 2019
AT1G08830	CSD1, superoxide dismutase [Cu-Zn] 1	superoxide anion radicals detoxification	cytoplasm, cytosol, nucleus	SUS	High	Kundu et al., 2013
AT4G25100	FSD1, superoxide dismutase [Fe] 1, chloroplastic	cytosolic, plastidic and nuclear iron superoxide dismutase, superoxide radicals detoxification	plastid, chloroplast stroma, nucleus, cytoplasm, apoplast	Y2H	High	Guo et al., 2019
AT2G25080	GPX1, phospholipid hydroperoxide glutathione peroxidase 1, chloroplastic	reduction of hydrogen peroxide, lipid peroxides and organic hydroperoxide by glutathione	plastid, chloroplast	Y2H	High	Guo et al., 2019
AT5G16710	DHAR3, glutathione-dependent dehydroascorbate reductase 3, chloroplastic	protein with dual function: glutathione-dependent thiol transferase and dehydroascorbate reductase activities	plastid, chloroplast	Y2H	High	Guo et al., 2019
AT1G76080	CDSP32, chloroplastic drought-induced stress protein of 32 KDa	putative thiol-disulfide oxidoreductase, alkyl hydroperoxides reduction	plastid, chloroplast	Y2H	High	Guo et al., 2019
AT1G20620	CAT3, catalase 3	hydrogen peroxide detoxification	peroxisome	Y2H	High	Guo et al., 2019
AT3G14420	GOX1, Glycolate oxidase 1	catalyzes the oxidation of glycolate to glyoxylate	peroxisome	Y2H	High	Guo et al., 2019

Y2H, yeast two hybrid assay; SUS, split ubiquitin system.

According to high-throughput Y2H PPI study, RACK1A may interact with cytosolic APX1 (Guo et al., 2019; **Table 4**). Noteworthy, both RACK1A and APX1 are involved in plant responses to ABA. *APX1* promoter possesses an ABA response element (ABRE; Saxena et al., 2020) and *APX1* is thought to be regulated by ABA. During simultaneous exposure to drought and heat stress, APX1 is likely responsible for regulation of H_2_O_2_ level important for stomata closure (Zandalinas et al., 2016). Additionally, *APX1* knock-out mutant exerts suppressed levels of *RACK1A* transcript in *Arabidopsis*, indicating the link between APX1 and RACK1A in H_2_O_2_ signaling pathways (Pnueli et al., 2003). These data indicate possible involvement of RACK1-APX1 interaction in plant ABA signaling.

RACK1 is also linked to the Cu^2+^-dependent regulation of SODs. *CSDs* and *FSD1* expressions are regulated in Cu^2+^-dependent manner *via* SPL7 and miR398 (Pilon et al., 2011). Interestingly, RACK1 isoforms are involved in the biogenesis of miR398, leading to negative regulation of *CSD1* and *CSD2* (Speth et al., 2013). This possibly occurs during salt stress conditions or ABA response, judging from the fact, that miRNA398 levels are decreased under salt stress conditions, resulting in enhanced *CSD1* and *CSD2* expression (Jagadeeswaran et al., 2009). The possible mechanistic link between RACK1A and FSD1 is supposed by the similar germination response of *fsd1* and *rack1a* mutants to salt stress (Guo et al., 2009; Dvořák et al., 2021a). However, RACK1 interacts with FSD1 and CSD1 also directly as revealed by high-throughput Y2H (FSD1) and low-throughput split-ubiquitin assay (CSD1; **Table 4**; **Table S1**; Kundu et al., 2013; Guo et al., 2019).

Previous high- and low-throughput Y2H, BiFC and pull-down interaction studies indicate that both RACK1A and FSD1 interact with components of G-protein complex. While RACK1A interacts with regulator of G-protein signaling 1(AtRGS1), AGB1 and guanine nucleotide-binding protein subunit gamma 1 and 2 (AGG1/2), FSD1 interacts with AtRGS1, GPA1 and AGB1 (Klopffleisch et al., 2011; Olejnik et al., 2011; Cheng et al., 2015). AtRGS1 accelerates the intrinsic GTP hydrolysis rate of GPA1, thus acting as a repressor of the active state of GPA1 (Chen et al., 2003). AtRGS1 was shown to be involved in glucose- and ABA-mediated signaling during seed germination and is responsible for the induction of ABA-responsive and ABA biosynthetic genes (Chen et al., 2006b).

The proposed interactions of RACK1A with other redox-related plastidic enzymes (glutathione peroxidase 1 (GPX1), DHAR3, chloroplastic drought-induced stress protein of 32 KDa (CDSP32) and peroxisomal enzymes (CAT3, glycolate oxidase 1 (GOX1); Guo et al., 2019) studied by high-throughput Y2H assay have not been validated and their function is not known (**Table 4**). RACK1A may not serve only as a scaffold mediating the interaction of other proteins during the redox responses, but may also stabilize the interaction partner and assist during its transport to the specific target organelle. In mouse melanocytes, interaction of VPS9-ankyrin repeat protein (VARP) with RACK1 leads to VARP stabilization (Marubashi et al., 2016). In human pulmonary artery endothelial cells, RACK1 interacts with TGF-β inhibited membrane-associated protein (TIMAP) and farnesyl transferase and ensures TIMAP prenylation and localization to the plasma membrane (Boratkó et al., 2013). It must be noted, that the reliability of *Arabidopsis* RACK1A PPIs with plastidic and peroxisomal proteins is questionable and they must be subjected to thorough validation.

A significant body of evidence shows that RACK1 is sensitive to oxidative/abiotic stress on mRNA and protein levels. Methyl viologen, a well-known oxidative stress inducer, enhanced the activity of the *RACK1A* promoter in *Arabidopsis* and overexpression of *RACK1* in rice led to increased sensitivity to this stress (Fennell et al., 2021). Soybean *GmRACK1* gene is downregulated by H_2_O_2_, suggesting its involvement in ROS signaling (Li et al., 2018). In addition, RACK1A homo-dimerizes under UV-B-induced oxidative stress as found by heterologous expression of *AtRACK1A* in yeast (Sabila et al., 2016). Notably, RACK1 protein may undergo redox PTMs. A redox proteomic study identified a reduction of conserved redox-sensitive cysteine residues in wheat RACK1 in response to ABA (Bykova et al., 2011). Specific redox-sensitive cysteine residues in the *Arabidopsis* RACK1A amino acid sequence can undergo S-nitrosylation (Cys138, Cys209; Hu et al., 2015), S-sulfenylation (Cys138, Cys 184; Huang et al., 2019) or S-sulfhydration (Cys138; Aroca et al., 2017), as proposed by proteomic studies. Bioinformatic modeling and predictions suggested that two of these cysteine residues (Cys138; Cys184) are redox-sensitive, have high surface accessibility (**Figure 1A**), and are conserved across plant kingdom (**Figure 1B**). These modifications may represent a possible regulatory mechanism by which RACK1 links external conditions with ROS homeostasis. Nevertheless, their physiological relevance remains unknown in plants. It may be expected that cysteine redox modifications modulate RACK1A function and have specific consequences in the plant stress tolerance. Oxidation of RACK1A may affect its localization, role in ABA signaling and MAPK scaffolding. In addition, it may also change the overall antioxidant capacity in the cell.

**Figure 2:**
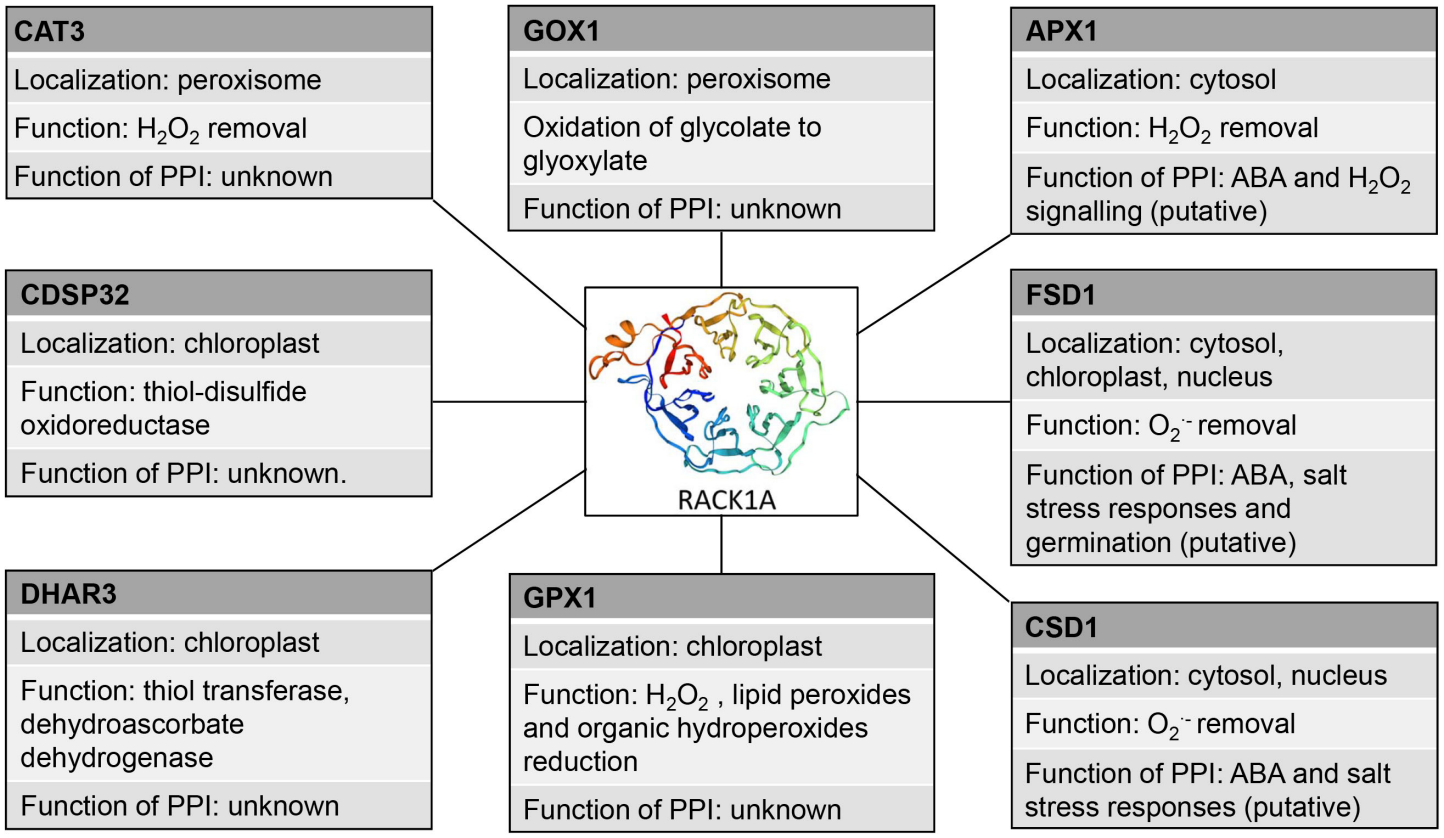
Schematic depiction of interaction partners of RACK1A with their antioxidant molecular functions and proposed functions of the interactions. PPI: protein-protein interaction, CAT3, catalase 3, GOX1 - Glycolate oxidase 1, APX1, ascorbate peroxidase 1, CDSP32 - chloroplastic drought-induced stress protein of 32 KDa, FSD1, iron superoxide dismutase, CSD1 Cu/Zn superoxide dismutase, GPX1, glutathuione peroxidase 1, DHAR3, dehydroascorbate reductase 3.

These data show that RACK1 may link cellular redox homeostasis under salt stress conditions or during ABA response to modulate antioxidant defense through PPIs (**Figure 2**). However, further functional studies are essential to support this hypothesis.

## Conclusion and future prospects

Our review shows that PPIs of antioxidant enzymes represent an important part of the machinery of ROS regulation. Through PPIs, these enzymes are regulated by folding, stabilization, degradation and activation, which have crucial consequences in ROS accumulation as well as plant stress tolerance. On the other hand, PPIs may link ROS scavenging with diverse metabolic and physiological processes. Importantly, proteins such as CDC48, LSDs and RACK1 may integrate regulation of multiple antioxidant enzymes, thus ensuring their synchronization and homeostasis. Nevertheless, PPIs in plant antioxidant defense are understudied and require further intensive research. It is of eminent importance to employ modern techniques of PPI identification combined with genetic and functional genetic studies. This should reveal the spatial and temporal orchestration of posttranslational, transcriptional and translational regulation of antioxidant enzymes employing PPIs.

## Author contributions

PM, PD and TT drafted the manuscript which was revised and edited by TT and JŠ. All authors approved the final version of the manuscript. All authors contributed to the article and approved the submitted version.
