# Supplementary material for: Protein-protein interactions in plant antioxidant defense
Source: Front Plant Sci. 2022 Dec 14;13:1035573. doi: 10.3389/fpls.2022.1035573 (PMC9795235; doi:10.3389/fpls.2022.1035573)
Supplement: Supplementary file 1 [file Table_1.pdf]

Protein-protein interactions in plant antioxidant defense

Pavol Melicher[1], Petr Dvořák[1], Jozef Šamaj[1], Tomáš Takáč[1*]

[1]Department of Biotechnology, Faculty of Science, Palacký University, Olomouc, Šlechtitelů 27, 783 71 Olomouc, Czech Republic

SUPPLEMENTARY TABLES

Table S1. List of interaction partners of *Arabidopsis thaliana* superoxide dismutases as found by high-throughput methods only, with their respective function and localization. CF – Co-fractionation; ER – endoplasmic reticulum; IP – immunopurification; MS – Mass spectrometry; PM – plasma membrane; TAP – Tandem affinity tag purification; SUS – Spilt-ubiquitin system; Y2H – Yeast two-hybrid assay; [1]Applies for FSD1 only.

Protein of interest	Interactor Accession	Interactor Name	Function	Localization	Method of detection	Reference
FSD1	AT4G34460	AGB1, guanine nucleotide-binding protein subunit beta	G-protein signaling	PM, cytoplasm, nucleus	Y2H	Klopffleisch et al., 2011
	AT4G14716	ARD2, acireductone dioxygenase 2	L-methionine biosynthesis via salvage pathway	cytoplasm, nucleus	Y2H	Klopffleisch et al., 2011
	AT5G23310	FSD3, chloroplastic	ROS scavenging, thylakoid membrane protection, chloroplast development	plastid, chloroplast thylakoid	Y2H	*Arabidopsis* Interactome Mapping Consortium, 2011
	AT2G26300	GPA1, guanine nucleotide-binding protein α-1 subunit	G-protein signaling	PM	Y2H	Klopffleisch et al., 2011
	AT2G16640	TOC132, translocase of chloroplast 132, chloroplastic	chloroplast-destined precursor protein translocation	plastid, chloroplast outer membrane, cytoplasm	SUS	Dutta et al., 2014
	AT3G60600	PVA11, vesicle-associated protein 1-1	component of the complex that forms a bridge between the ER and PM	ER membrane, tonoplast	Y2H	Klopffleisch et al., 2011
	AT1G18080	RACK1A, receptor for activated C kinase 1A	scaffold protein, regulation of signal transduction	cytoplasm, nucleus	SUS	Guo et al., 2019

	AT3G26090	RGS1, regulator of G-protein signaling 1	D-glucose receptor, GTPase activity regulation, hormonal signaling	PM, endosome membrane	Y2H	Klopffleisch et al., 2011
FSD1/2	AT5G03240	UBQ3, polyubiquitin 3	protein degradation	cytoplasm, nucleus	IP-MS[1]<br>TAP-MS	Igawa et al., 2009<br>Kim et al., 2013
FSD2	AT1G69690	TCP15, transcription factor	hormonal responses, plant development, endoreduplication, pathogenesis-related protein 2 expression	nucleus, chloroplast	Y2H	*Arabidopsis* Interactome Mapping Consortium, 2011
	AT3G02150	TCP13, transcription factor	negative regulator of boundary-specific genes expression, leaf differentiation.	nucleus, chloroplast	Y2H	*Arabidopsis* Interactome Mapping Consortium, 2011
	AT2G19650	cysteine/histidine-rich C1 domain family protein	Unknown	Unknown	Y2H	*Arabidopsis* Interactome Mapping Consortium, 2011
	AT4G10800	BTB/POZ domain protein	probable substrate-specific adapter of an E3 ubiquitin-protein ligase complex	Unknown	Y2H	*Arabidopsis* Interactome Mapping Consortium, 2011
	AT2G39760	BPM3, BTB/POZ and MATH domain-containing protein 3	probable substrate-specific adaptor of an E3 ubiquitin-protein ligase complex	Unknown	Y2H	Altmann et al., 2020
	AT5G50870	UBC27, ubiquitin-conjugating enzyme 27	covalent attachment of ubiquitin	nucleus	Y2H	*Arabidopsis* Interactome Mapping Consortium, 2011
FSD3	AT3G54290	AT3G54290, hemerythrin domain-containing protein	Unknown	Unknown	Y2H	*Arabidopsis* Interactome Mapping Consortium, 2011
	AT5G40590	cysteine/histidine-rich C1 domain family protein	Unknown	Unknown	Y2H	*Arabidopsis* Interactome Mapping Consortium, 2011
CSD1	AT1G71050	HIPP20, heavy metal-associated isoprenylated plant protein 20	metallochaperone, heavy metal homeostasis and detoxification	membrane	Y2H	*Arabidopsis* Interactome Mapping Consortium, 2011
CSD1/3	AT5G03240	UBQ3, polyubiquitin 3	protein degradation	cytoplasm, nucleus	TAP-MS	Kim et al., 2013
CSD1/2	AT3G21630	CERK1, chitin elicitor receptor kinase 1	receptor-like kinase, innate immunity	PM	Y2H	Le et al., 2014
CSD2	AT3G26090	RGS1, regulator of G-protein signaling 1	D-glucose receptor, involved in GTPase activity regulation, hormonal signaling	cell membrane, endosome membrane	Y2H	Klopffleisch et al., 2011
MSD1	AT4G16760	ACX1, peroxisomal acyl-coenzyme A oxidase 1	fatty acid beta-oxidation, jasmonate biosynthesis	peroxisome	CF-MS	McWhite et al., 2020
	AT2G35690	ACX1.2, putative peroxisomal acyl-coenzyme A oxidase 1.2	fatty acid beta-oxidation	peroxisome	CF-MS	McWhite et al., 2020
	AT5G19550	ASP2, aspartate aminotransferase isozyme 1	metabolism of amino acids and Krebs-cycle related organic acids	cytoplasm	CF-MS	McWhite et al., 2020

AT5G11520	ASP3, aspartate aminotransferase 3, chloroplastic	metabolism of amino acids and Krebs cycle-related organic acids	plastid, chloroplast	CF-MS	McWhite et al., 2020
AT5G08300	SCS-alpha-1, succinate--CoA ligase [ADP-forming] subunit alpha-1, mitochondrial	citrate cycle	mitochondrion	CF-MS	McWhite et al., 2020
AT5G23250	SCS-alpha-2, succinate--CoA ligase [ADP-forming] subunit alpha-2, mitochondrial	citrate cycle	mitochondrion	CF-MS	McWhite et al., 2020
AT4G03205	HEMF2, coproporphyrinogen-III oxidase 2, chloroplastic	heme biosynthesis	plastid, chloroplast	CF-MS	McWhite et al., 2020
AT1G03475	HEMF1, coproporphyrinogen-III oxidase 1, chloroplastic	heme biosynthesis	plastid, chloroplast	CF-MS	McWhite et al., 2020
AT3G22200	POP2, gamma-aminobutyrate transaminase, mitochondrial	gamma-aminobutyric acid metabolism, shoot apical meristem maintenance	mitochondrion	CF-MS	McWhite et al., 2020
AT1G53240	mMDH1, malate dehydrogenase 1	citrate cycle, photorespiration	mitochondrion	CF-MS	McWhite et al., 2020
AT3G15020	mMDH2, malate dehydrogenase 2	citrate cycle, photorespiration	mitochondrion	CF-MS	McWhite et al., 2020
AT2G20420	SCS-beta, succinate--CoA ligase [ADP-forming] subunit beta	citrate cycle	mitochondrion	CF-MS	McWhite et al., 2020
AT2G21170	TIM, triosephosphate isomerase, chloroplastic	sugar metabolism, Calvin cycle	plastid, chloroplast	CF-MS	McWhite et al., 2020
AT3G55440	TPI, triosephosphate isomerase, cytosolic	sugar metabolism	cytoplasm, mitochondrion	CF-MS	McWhite et al., 2020

Table S2. List of interaction partners of *Arabidopsis thaliana* catalases found by high-throughput methods only, with their respective function and localization. ABA – abscisic acid; AP – Affinity purification; IP – immunopurification; MS – Mass spectrometry; PM – plasma membrane; TAP – Tandem affinity tag purification; SUS – Spilt-ubiquitin system; Y2H – Yeast two-hybrid assay.

**Protein of interest**	**Accession**	**Interactor Name**	**Function**	**Localization**	**Method of detection**	**Reference**
CAT1	AT3G18140	LST8-1, target of rapamycin complex subunit	plant growth, ABA accumulation	endosome	TAP-MS	Van Leene et al., 2019
	AT5G67530	CYP65, peptidyl-prolyl cis-trans isomerase-like 2	protein folding, protein transport, probable ubiquitin ligase activity	nucleus	Y2H	*Arabidopsis* Interactome Mapping Consortium, 2011
	AT3G08850	RAPTOR1, regulatory-associated protein of TOR 1	putative component of TOR kinase pathway, osmotic stress response	cytoplasm	TAP-MS	Van Leene et al., 2019
CAT1/2	AT3G49250	DMS3, defective in meristem silencing 3	gene silencing	nucleus	Y2H	*Arabidopsis* Interactome Mapping Consortium, 2011
CAT1/2/3	AT4G30840	NUP43, nuclear pore complex protein	protein and mRNA nuclear transport	nuclear pore complex	IP-MS	Tamura et al., 2010
CAT2	AT2G01950	BRL2, serine/threonine-protein kinase BRI1-like 2	receptor, serine/threonine-protein kinase activity, differentiation of provascular and procambial cells	PM	interaction cloning	Ceserani et al., 2009
	AT1G78300	GRF2, 14-3-3-like protein GF14 omega	associated with complex that binds to the G box of DNA	cytoplasm, nucleus	TAP-MS	Chang et al., 2009
CAT3	AT2G41620	NUP93A, nuclear pore complex protein	nuclear transport	nuclear pore complex	IP-MS	Tamura et al., 2010
	AT3G14980	IDM1, increased DNA methylation 1	histone H3 acetyltransferase, histone acetylation	nucleus	AP-MS	Li et al., 2015b
	AT1G18080	RACK1A, receptor for activated C kinase 1A	scaffold protein, regulation of signal transduction	cytoplasm, nucleus	SUS	Guo et al., 2019
	AT5G38480	GRF3, 14-3-3-like protein GF14 psi	associated with complex that binds to the G box of DNA	cytoplasm, nucleus	AP-MS	Shin et al., 2010
	AT5G03240	UBQ3, polyubiquitin 3	protein degradation	cytoplasm, nucleus	IP-MS	Igawa et al., 2009
	AT1G80670	RAE1, RNA export factor 1	nuclear transport	nuclear pore complex	IP-MS	Tamura et al., 2010

Table S3. List of interaction partners of *Arabidopsis thaliana* ascorbate-glutathione cycle enzymes found by high-throughput methods only, with their respective function and localization. AP – Affinity purification; ER – endoplasmic reticulum; GABA – gamma aminobutyric acid; IP – immunopurification; MS – Mass spectrometry; PM – plasma membrane; TAP – Tandem affinity tag purification; SUS – Spilt-ubiquitin system; mbSUS – mating-based SUS; Y2H – Yeast two-hybrid assay.

**Protein of interest**	**Interactor Accession**	**Name**	**Function**	**Localization**	**Method of detection**	**Reference**
APX1	AT1G15570	CYCA2;3, nucleolar A2-type cyclin CYCA2.3	endoreduplication and endocycles	nucleus	TAP-MS	Boudolf et al., 2009
					TAP-MS	Van Leene et al., 2010
	AT3G21630	CERK1, chitin elicitor receptor kinase 1	receptor-like kinase, innate immunity	PM	Y2H	Le et al., 2014
	AT3G24300	AMT1;3, ammonium transporter 1;3	ammonium translocation	PM	IP-MS	Bellati et al., 2016
	AT4G19360	SCD6-related protein	unknown	unknown	Y2H	*Arabidopsis* Interactome Mapping Consortium, 2011
	AT2G45960	PIP1B, aquaporin PIP1-2	water transport, transport of small solutes; hydraulics, carbon fixation, plant development	PM	IP-MS	Bellati et al., 2016
	AT3G53420	PIP2A, aquaporin PIP2-1	water transport; transport of small solutes	PM	IP-MS	Bellati et al., 2016
	AT3G51030	TRX1, thioredoxin H1	redox regulation, activation of cytosolic malate dehydrogenase	cytoplasm	AP-MS	Ueoka-Nakanishi et al., 2013
	AT5G42980	TRX3, thioredoxin H3	redox regulation	cytoplasm	*in vitro* protein reduction/ MS	Marchand et al., 2004
	AT1G18080	RACK1A, receptor for activated C kinase 1A	scaffold protein, regulation of signal transduction	cytoplasm, nucleus	SUS	Guo et al., 2019
	AT5G03240	UBQ3, polyubiquitin 3	protein degradation	cytoplasm, nucleus	IP-MS	Igawa et al., 2009
					TAP-MS	Kim et al., 2013
APX6	AT3G26090	RGS1, regulator of G-protein signaling 1	D-glucose receptor, GTPase activity regulation, hormonal signaling	PM, endosome membrane	Y2H	Klopffleisch et al., 2011
MDAR1	AT5G43980	PDLP1, plasmodesmata-located protein 1	cell-to-cell trafficking, systemic acquired resistance	PM, plasmodesma	Co-IP-MS	Caillaud et al., 2014

MDAR1/2/3/6	AT1G70730	PGM2, phosphoglucomutase 2	glucose metabolism	cytoplasm	CF-MS	McWhite et al., 2020
	AT1G23190	PGM3, phosphoglucomutase 3	glucose metabolism	cytoplasm	CF-MS	McWhite et al., 2020
	AT5G13420	TRA2, transaldolase 2	pentose-phosphate pathway	plastid, chloroplast stroma	CF-MS	McWhite et al., 2020
	AT4G09320	NDPK1, nucleoside diphosphate kinase 1	NTP synthesis, oxidative stress	peroxisome, nucleus, cytoplasm	CF-MS	McWhite et al., 2020
	AT3G12780	PGK1, phosphoglycerate kinase 1	sugar metabolism, galactolipid biosynthesis	plastid, chloroplast	CF-MS	McWhite et al., 2020
	AT1G56190	PGK2, phosphoglycerate kinase 2	sugar metabolism, galactolipid biosynthesis	plastid, chloroplast	CF-MS	McWhite et al., 2020
	AT1G79550	PGK3, phosphoglycerate kinase 3	sugar metabolism	plastid, chloroplast	CF-MS	McWhite et al., 2020
	AT5G17310	UGP1, UTP--glucose-1-phosphate uridylyltransferase 1	polysaccharide synthesis	cytoplasm	CF-MS	McWhite et al., 2020
	AT3G03250	UGP2, UTP--glucose-1-phosphate uridylyltransferase 2	polysaccharide synthesis	cytoplasm	CF-MS	McWhite et al., 2020
	AT1G53240	mMDH1, malate dehydrogenase 1, mitochondrial	citrate cycle, photorespiration	mitochondrion matrix	CF-MS	McWhite et al., 2020
	AT3G15020	mMDH2, malate dehydrogenase 2, mitochondrial	citrate cycle, photorespiration	mitochondrion matrix	CF-MS	McWhite et al., 2020
	AT2G20420	SCS-beta, succinate--CoA ligase [ADP-forming] subunit beta	citrate cycle	mitochondrion	CF-MS	McWhite et al., 2020
	AT2G21170	TIM, triosephosphate isomerase, chloroplastic	sugar metabolism, Calvin cycle	plastid, chloroplast	CF-MS	McWhite et al., 2020
	AT3G55440	TPI, triosephosphate isomerase, cytosolic	sugar metabolism	cytoplasm, mitochondrion	CF-MS	McWhite et al., 2020
MDAR2	AT3G17240	mtLPD2, dihydrolipoyl dehydrogenase 2, mitochondrial	aminoacid metabolism	mitochondrion matrix	AP-MS	Zhang et al., 2018
MDAR2/6	AT1G43560	AtTrxy2, thioredoxin Y2, chloroplastic	redox regulation, chloroplastic malate dehydrogenase and fructose-1,6-bisphosphatase activation	plastid, chloroplast stroma	AP-MS	Marchand et al., 2010
MDAR6	AT2G15620	NIR1, ferredoxin--nitrite reductase 1	nitrate assimilation	plastid, chloroplast	Y2H	*Arabidopsis* Interactome Mapping Consortium, 2011
	AT5G03240	UBQ3, polyubiquitin 3	protein degradation	cytoplasm, nucleus	IP-MS	Igawa et al., 2009
					TAP-MS	Kim et al., 2013

DHAR1 (mitochondrial)	AT1G33490	E3 ubiquitin-protein ligase	protein degradation	unknown	mbSUS	Jones et al., 2014
	AT3G23560	DTX19, protein detoxification 19	transmembrane transport	membrane	mbSUS	Jones et al., 2014
	AT1G33100	DTX20, protein detoxification 20	transmembrane transport	membrane	mbSUS<br>split-GFP	Jones et al., 2014
	AT1G71140	DTX14, protein detoxification 14	transmembrane transport	membrane	mbSUS	Jones et al., 2014
	AT1G47530	DTX33, protein detoxification 33	transmembrane transport	membrane	mbSUS	Jones et al., 2014
	AT5G49130	DTX55, protein detoxification 55	transmembrane transport	membrane	mbSUS	Jones et al., 2014
	AT2G28960	AT2G28960, putative LRR receptor-like serine/threonine-protein kinase	signaling	membrane	mbSUS	Jones et al., 2014
	AT2G41820	PCX3, Leucine-rich repeat receptor-like tyrosine-protein kinase	signaling	PM	mbSUS	Jones et al., 2014
	AT1G29330	ERD2, ER lumen protein-retaining receptor 2	protein transport, vesicular traffic through the Golgi	ER membrane	mbSUS	Jones et al., 2014
	AT3G05360	RLP30, receptor like protein 30	receptor, non-host resistance to bacterial pathogens	cell membrane	mbSUS	Jones et al., 2014
	AT5G61570	protein kinase-like protein	Unknown	Unknown	mbSUS	Jones et al., 2014
	AT5G48380	BIR1, BAK1-interacting receptor-like kinase	signaling, pathogen response	cell membrane	mbSUS	Jones et al., 2014
	AT2G23070	CKA4, casein kinase II subunit alpha-4	ABA signaling, retrograde signaling	plastid, chloroplast	mbSUS	Jones et al., 2014
	AT3G46370	AT3G46370, leucine-rich repeat protein kinase-like protein	unknown	unknown	mbSUS	Jones et al., 2014
	AT5G38660	APE1, acclimation of photosynthesis to environment	photosynthesis	plastid, chloroplast	mbSUS	Jones et al., 2014
	AT1G05640	AT1G05640, ankyrin repeats-containing protein	unknown	membrane	mbSUS	Jones et al., 2014
	AT1G11450	AT1G11450, WAT1-related protein	transmembrane transport	membrane	mbSUS	Jones et al., 2014
	AT1G52550	AT1G52550, hypothetical protein	unknown	unknown	mbSUS	Jones et al., 2014
	AT2G23093	AT2G23093, major facilitator superfamily protein	unknown	unknown	mbSUS	Jones et al., 2014
	AT2G28315	UXT1, UDP-xylose transporter 1	nucleotide/sugar transmembrane transport	Golgi apparatus membrane, ER membrane	mbSUS	Jones et al., 2014

AT2G31790	UGT74C1, UDP-glycosyltransferase 74C1	sugar metabolism	unknown	Y2H	*Arabidopsis* Interactome Mapping Consortium, 2011
AT2G39060	SWEET9, bidirectional sugar transporter	nectar transmembrane transport	PM, vesicle membrane, Golgi apparatus, trans-Golgi network membrane	mbSUS	Jones et al., 2014
AT2G42390	protein kinase C substrate, heavy chain-like protein	unknown	unknown	mbSUS	Jones et al., 2014
AT3G16180	NTR1.12, nitrate transporter 1.12	nitrate transmembrane transport	PM	mbSUS	Jones et al., 2014
AT3G52640	nicastrin	gamma-secretase complex protein	membrane	mbSUS	Jones et al., 2014
AT3G59090	CAND3, candidate G-protein coupled receptor 3	tobamovirus multiplication protein	membrane	mbSUS	Jones et al., 2014
AT3G59780	rhodanese/cell cycle control phosphatase superfamily protein	cell cycle	unknown	mbSUS	Jones et al., 2014
AT4G16444	GET1, guided entry of tail-anchored proteins 1	transmembrane transport to ER	ER membrane	mbSUS	Jones et al., 2014
AT4G19645	AT4G19645, TRAM, LAG1 and CLN8 (TLC) lipid-sensing domain containing protein	unknown	membrane	mbSUS	Jones et al., 2014
AT4G30660	UPF0057 membrane protein	unknown	membrane	mbSUS	Jones et al., 2014
AT4G31340	Uncharacterized protein	unknown	unknown	mbSUS	Jones et al., 2014
AT5G01960	RING/U-box superfamily protein	unknown	membrane	mbSUS	Jones et al., 2014
AT5G19130	AT5G19130, GPI transamidase component family protein / Gaa1-like family protein	unknown	unknown	mbSUS	Jones et al., 2014
AT5G40670	Cystinosin homolog	putative L-cystine transmembrane transporter	lysosome membrane	mbSUS	Jones et al., 2014
AT5G41800	GAT2, putative GABA transporter 2	putative GABA transmembrane transporter	PM	mbSUS	Jones et al., 2014
AT5G47530	AT5G47530, cytochrome b561 and DOMON domain-containing protein	putative catecholamine-responsive trans-membrane electron transporter	membrane	mbSUS	Jones et al., 2014
AT4G39350	CESA2, cellulose synthase A catalytic subunit 2 [UDP-forming]	cellulose synthesis and cell wall formation	PM	mbSUS	Jones et al., 2014

	AT5G01690	CHX27, cation/H(+) antiporter 27	putative Na^+/H^+ antiporter	membrane	mbSUS	Jones et al., 2014
	AT2G29090	CYP707A2, abscisic acid 8'-hydroxylase 2	ABA degradation	membrane	mbSUS	Jones et al., 2014
	AT1G78490	CYP708A3, cytochrome P450, family 708, subfamily A, polypeptide 3	unknown	unknown	mbSUS	Jones et al., 2014
	AT3G23410	FAO3, long-chain-alcohol oxidase	omega-oxidation pathway of lipid degradation	membrane	mbSUS	Jones et al., 2014
	AT5G16150	GLT1, glucose transporter 1	efflux of glucose to cytosol	plastid, chloroplast inner membrane	mbSUS	Jones et al., 2014
	AT3G01550	PPT2, phosphoenolpyruvate/phosphate translocator 2	phosphoenolpyruvate/phosphate translocator	plastid, chloroplast membrane	mbSUS	Jones et al., 2014
	AT2G19580	TET2, tetraspanin-2	cell differentiation	membrane	mbSUS	Jones et al., 2014
	AT1G05680	UGT74E2, uridine diphosphate glycosyltransferase 74E2	glucosyltransferase, auxin signaling	unknown	Y2H	*Arabidopsis* Interactome Mapping Consortium, 2011
	AT1G15610	hypothetical protein	unknown	unknown	mbSUS	Jones et al., 2014
	AT5G40640	hypothetical protein	unknown	unknown	mbSUS	Jones et al., 2014
	AT1G15900	hypothetical protein	unknown	unknown	mbSUS	Jones et al., 2014
	AT5G03240	UBQ3, polyubiquitin 3	protein degradation	cytoplasm, nucleus	IP-MS	Igawa et al., 2009
					TAP-MS	Kim et al., 2013
DHAR2	AT1G43560	AtTrxy2, thioredoxin Y2, chloroplastic	redox regulation, chloroplastic malate dehydrogenase and fructose-1,6-bisphosphatase activation	plastid, chloroplast stroma	AP-MS	Marchand et al., 2010
DHAR3	AT1G18080	RACK1A, receptor for activated C kinase 1A	scaffold protein, signal transduction	cytoplasm, nucleus	SUS	Guo et al., 2019
	AT5G42980	TRX3, thioredoxin H3	redox regulation	cytoplasm	*in vitro* protein reduction/MS	Marchand et al., 2004
GR1/2	AT3G23940	DHAD, dihydroxy-acid dehydratase	biosynthesis of branched-chain amino acids	plastid, chloroplast	CF-MS	McWhite et al., 2020
	AT4G33030	SQD1, sulfoquinovosyldiacylglycerol 1	biosynthesis of sulfolipids	plastid, chloroplast	CF-MS	McWhite et al., 2020
	AT5G49970	PPOX1, pyridoxine/pyridoxamine 5'-phosphate oxidase 1	pyridoxal 5'-phosphate metabolism	plastid, chloroplast	CF-MS	McWhite et al., 2020

AT1G21750	PDIL1-1, protein disulfide isomerase-like 1-1	rearrangement of -S-S- bonds in proteins, programmed cell death	ER lumen, vacuole	CF-MS	McWhite et al., 2020
AT1G77510	PDIL1-2, protein disulfide isomerase-like 1-2	rearrangement of -S-S- bonds in proteins	ER lumen	CF-MS	McWhite et al., 2020
AT1G48850	EMB1144, chorismate synthase, chloroplastic	chorismate biosynthesis, biosynthesis of aromatic amino acids	plastid, chloroplast	CF-MS	McWhite et al., 2020
AT5G17380	HACL, 2-hydroxyacyl-CoA lyase	catalysis of carbon-carbon cleavage	Unknown	CF-MS	McWhite et al., 2020
AT3G62510	protein disulfide isomerase-like protein	Unknown	Unknown	CF-MS	McWhite et al., 2020
AT3G60750	TKL1, transketolase 1	sugar metabolism, Calvin cycle	plastid, chloroplast stroma	CF-MS	McWhite et al., 2020
AT2G45290	TKL2, transketolase 2	sugar metabolism, Calvin cycle	plastid, chloroplast thylakoid membrane, chloroplast stroma	CF-MS	McWhite et al., 2020
AT4G33090	APM1, aminopeptidase M1	seedling development, cell cycle progression	membrane, microsome membrane, cytoplasm	CF-MS	McWhite et al., 2020

Supplementary References

Altmann, M., Altmann, S., Rodriguez, P.A. et al. (2020). Extensive signal integration by the phytohormone protein network. *Nature* 583, 271–276 (2020). https://doi.org/10.1038/s41586-020-2460-0

Arabidopsis Interactome Mapping Consortium (2011). Evidence for network evolution in an Arabidopsis interactome map. *Science* 333, 601–607. doi: 10.1126/science.1203877.

Bellati, J., Champeyroux, C., Hem, S., Rofidal, V., Krouk, G., Maurel, C., et al. (2016). Novel aquaporin regulatory mechanisms revealed by interactomics. *Mol. Cell. Proteomics* 15, 3473–3487. doi: 10.1074/mcp.M116.060087.

Boudolf, V., Lammens, T., Boruc, J., Van Leene, J., Van Den Daele, H., Maes, S., et al. (2009). CDKB1;1 forms a functional complex with CYCA2;3 to suppress endocycle onset. *Plant Physiol.* 150, 1482–1493. doi: 10.1104/pp.109.140269.

Caillaud, M.-C., Wirthmueller, L., Sklenar, J., Findlay, K., Piquerez, S. J. M., Jones, A. M. E., et al. (2014). The plasmodesmal protein PDLP1 localises to haustoria-associated membranes during downy mildew infection and regulates callose deposition. *PLoS Pathog.* 10, e1004496. doi: 10.1371/journal.ppat.1004496.

Ceserani, T., Trofka, A., Gandotra, N., and Nelson, T. (2009). VH1/BRL2 receptor-like kinase interacts with vascular-specific adaptor proteins VIT and VIK to influence leaf venation. *Plant J.* 57, 1000–1014. doi: 10.1111/j.1365-313X.2008.03742.x.

Chang, I.-F., Curran, A., Woolsey, R., Quilici, D., Cushman, J., Mittler, R., et al. (2009). Proteomic profiling of tandem affinity purified 14-3-3 protein complexes in Arabidopsis thaliana. *Proteomics* 9, 2967–2985. doi: 10.1002/pmic.200800445.

Dutta, S., Teresinski, H. J., and Smith, M. D. (2014). A split-ubiquitin yeast two-hybrid screen to examine the substrate specificity of atToc159 and atToc132, two Arabidopsis chloroplast preprotein import receptors. *PLoS One* 9, e95026. doi: 10.1371/journal.pone.0095026.

Guo, J., Hu, Y., Zhou, Y., Zhu, Z., Sun, Y., Li, J., et al. (2019). Profiling of the Receptor for Activated C Kinase 1a (RACK1a) interaction network in Arabidopsis thaliana. *Biochem. Biophys. Res. Commun.* 520, 366–372. doi: 10.1016/j.bbrc.2019.09.142.

Igawa, T., Fujiwara, M., Takahashi, H., Sawasaki, T., Endo, Y., Seki, M., et al. (2009). Isolation and identification of ubiquitin-related proteins from Arabidopsis seedlings. *J. Exp. Bot.* 60, 3067–3073. doi: 10.1093/jxb/erp134.

Jones, A. M., Xuan, Y., Xu, M., Wang, R.-S., Ho, C.-H., Lalonde, S., et al. (2014). Border control--a membrane-linked interactome of Arabidopsis. *Science* 344, 711–716. doi: 10.1126/science.1251358.

Kim, D.-Y., Scalf, M., Smith, L. M., and Vierstra, R. D. (2013). Advanced proteomic analyses yield a deep catalog of ubiquitylation targets in Arabidopsis. *Plant Cell* 25, 1523–1540. doi: 10.1105/tpc.112.108613.

Klopffleisch, K., Phan, N., Augustin, K., Bayne, R. S., Booker, K. S., Botella, J. R., et al. (2011). Arabidopsis G-protein interactome reveals connections to cell wall carbohydrates and morphogenesis. *Mol. Syst. Biol.* 7, 532. doi: 10.1038/msb.2011.66.

Le, M. H., Cao, Y., Zhang, X.-C., and Stacey, G. (2014). LIK1, a CERK1-interacting kinase, regulates plant immune responses in Arabidopsis. *PLoS One* 9, e102245. doi: 10.1371/journal.pone.0102245.

Li, Q., Wang, X., Sun, H., Zeng, J., Cao, Z., Li, Y., et al. (2015b). Regulation of Active DNA Demethylation by a Methyl-CpG-Binding Domain Protein in Arabidopsis thaliana. *PLoS Genet.* 11, e1005210. doi: 10.1371/journal.pgen.1005210.

Marchand, C. H., Vanacker, H., Collin, V., Issakidis-Bourguet, E., Maréchal, P. L., and Decottignies, P. (2010). Thioredoxin targets in Arabidopsis roots. *Proteomics* 10, 2418–2428. doi: 10.1002/pmic.200900835.

Marchand, C., Le Maréchal, P., Meyer, Y., Miginiac-Maslow, M., Issakidis-Bourguet, E., and Decottignies, P. (2004). New targets of Arabidopsis thioredoxins revealed by proteomic analysis. *Proteomics* 4, 2696–2706. doi: 10.1002/pmic.200400805.

McWhite, C. D., Papoulas, O., Drew, K., Cox, R. M., June, V., Dong, O. X., et al. (2020). A Pan-plant protein complex map reveals deep conservation and novel assemblies. *Cell* 181, 460-474.e14. doi: 10.1016/j.cell.2020.02.049.

Shin, R., Jez, J. M., Basra, A., Zhang, B., and Schachtman, D. P. (2011). 14-3-3 proteins fine-tune plant nutrient metabolism. *FEBS Lett.* 585, 143–147. doi: 10.1016/j.febslet.2010.11.025.

Tamura, K., Fukao, Y., Iwamoto, M., Haraguchi, T., and Hara-Nishimura, I. (2010). Identification and characterization of nuclear pore complex components in Arabidopsis thaliana. *Plant Cell* 22, 4084–4097. doi: 10.1105/tpc.110.079947.

Ueoka-Nakanishi, H., Sazuka, T., Nakanishi, Y., Maeshima, M., Mori, H., and Hisabori, T. (2013). Thioredoxin h regulates calcium dependent protein kinases in plasma membranes. *FEBS J.* 280, 3220–3231. doi: 10.1111/febs.12301.

Van Leene, J., Han, C., Gadeyne, A., Eeckhout, D., Matthijs, C., Cannoot, B., et al. (2019). Capturing the phosphorylation and protein interaction landscape of the plant TOR kinase. *Nat. Plants* 5, 316–327. doi: 10.1038/s41477-019-0378-z.

Van Leene, J., Hollunder, J., Eeckhout, D., Persiau, G., Van De Slijke, E., Stals, H., et al. (2010). Targeted interactomics reveals a complex core cell cycle machinery in Arabidopsis thaliana. *Mol. Syst. Biol.* 6, 397. doi: 10.1038/msb.2010.53.

Zhang, Y., Swart, C., Alseekh, S., Scossa, F., Jiang, L., Obata, T., et al. (2018). The extra-pathway interactome of the TCA cycle: Expected and unexpected metabolic interactions. *Plant Physiol.* 177, 966–979. doi: 10.1104/pp.17.01687.
